# Consensus holistic virtual screening for drug discovery: a novel machine learning model approach

**DOI:** 10.1186/s13321-024-00855-8

**Published:** 2024-05-28

**Authors:** Said Moshawih, Zhen Hui Bu, Hui Poh Goh, Nurolaini Kifli, Lam Hong Lee, Khang Wen Goh, Long Chiau Ming

**Affiliations:** 1 PAPRSB Institute of Health Sciences, Universiti Brunei Darussalam, Gadong, Brunei Darussalam; 2 Faculty of Computing and Engineering, Quest International University, Ipoh, Malaysia; 3 Faculty of Data Science and Information Technology, INTI International University, Nilai, Malaysia; 4 School of Medical and Life Sciences, Sunway University, Sunway City, Malaysia

**Keywords:** Consensus scoring, Virtual screening, Machine learning models, QSAR, Docking, Pharmacophore, Shape similarity

## Abstract

In drug discovery, virtual screening is crucial for identifying potential hit compounds. This study aims to present a novel pipeline that employs machine learning models that amalgamates various conventional screening methods. A diverse array of protein targets was selected, and their corresponding datasets were subjected to active/decoy distribution analysis prior to scoring using four distinct methods: QSAR, Pharmacophore, docking, and 2D shape similarity, which were ultimately integrated into a single consensus score. The fine-tuned machine learning models were ranked using the novel formula “w_new”, consensus scores were calculated, and an enrichment study was performed for each target. Distinctively, consensus scoring outperformed other methods in specific protein targets such as PPARG and DPP4, achieving AUC values of 0.90 and 0.84, respectively. Remarkably, this approach consistently prioritized compounds with higher experimental PIC_50_ values compared to all other screening methodologies. Moreover, the models demonstrated a range of moderate to high performance in terms of R^2^ values during external validation. In conclusion, this novel workflow consistently delivered superior results, emphasizing the significance of a holistic approach in drug discovery, where both quantitative metrics and active enrichment play pivotal roles in identifying the best virtual screening methodology.

**Scientific contribution**

We presented a novel consensus scoring workflow in virtual screening, merging diverse methods for enhanced compound selection. We also introduced ‘w_new’, a groundbreaking metric that intricately refines machine learning model rankings by weighing various model-specific parameters, revolutionizing their efficacy in drug discovery in addition to other domains.

**Graphical Abstract:**

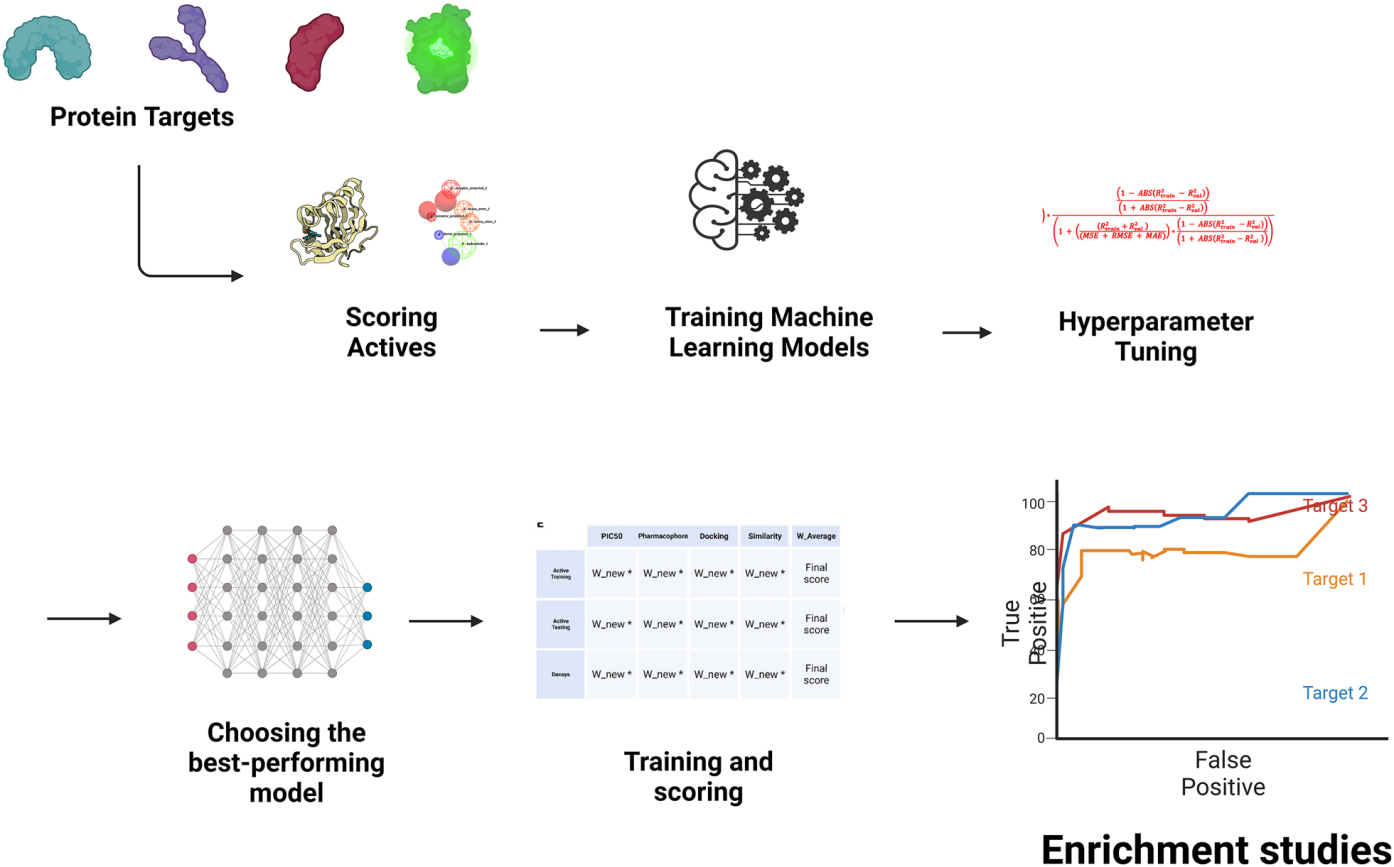

**Supplementary Information:**

The online version contains supplementary material available at 10.1186/s13321-024-00855-8.

## Introduction

In the realm of modern drug discovery, virtual screening stands as a pivotal cornerstone [1]. This computational strategy serves as the beacon for researchers, directing them through vast chemical libraries to efficiently uncover potential drug candidates [2]. As elucidated by Baber, Shirley [3], there exists a burgeoning interest in consensus approaches tailored explicitly for ligand-based virtual screening. Such approaches are not mere theoretical constructs; they are the culmination of intricate combinations of multiple properties, each contributing a unique facet to the screening process. Among the properties integrated into these consensus approaches are structural, 2D pharmacophore, and property-based fingerprints. Additionally, scores derived using BCUT descriptors, an Eigenvalue-based molecular descriptor [4], and 3D pharmacophore methods further enhance the screening's breadth and depth [5]. Consensus scoring enhances data set enrichment over single scoring functions by approximating the true value more closely through repeated samplings akin to multiple scoring functions, improving active compound clustering thereby recovering more actives than decoys [3].

Exploring the methodologies employed in consensus docking programs, Houston and Walkinshaw [6] introduced consensus docking as a method to enhance the accuracy of pose prediction in virtual screening by combining the results from multiple docking programs. The study tested Autodock [7], DOCK [8], and Vina [9], finding that while individual success rates for accurate pose prediction ranged from 55 to 64%, using a consensus approach increased this accuracy to over 82%. This method reduces false positives by advancing only those compounds to the scoring stage that are similarly docked by multiple programs, thereby improving the efficiency of virtual screening and the likelihood of identifying viable drug candidates. Consensus molecular docking workflows are regarded as critical methodologies within virtual screening approaches, primarily aimed at enhancing the identification of genuine actives during virtual screening campaigns [10–12]. But the exploration doesn't halt in consensus docking software.

Additional studies delve into the intricate tapestry of virtual screening methodologies, uncovering both sequential [13] and parallel [14] approaches. Sequential approaches, as the name suggests, unfold in a stepwise manner, systematically applying various techniques on a progressively decreasing number of compounds. This meticulous workflow encompasses stages such as pharmacophore screening, judicious application of property filters, followed by docking, culminating in manual selection. In stark contrast, parallel approaches deploy a multitude of methods independently but on a consistent number of compounds. Techniques such as pharmacophores, similarity methods, and docking are executed simultaneously, culminating in a robust automated selection process [15, 16]. In a bid to augment virtual screening's precision, researchers introduce a novel probabilistic paradigm. This framework, meticulously crafted to combine structure- and ligand-based screening methods to improve the accuracy of virtual screening predictions by fusing them into robust probabilities of activity, providing a quantitative bioactivity likelihood for compounds, thereby enhancing predictions [17].

Navigating further into the heart of the virtual screening, a comprehensive exploration of traditional consensus scoring unfolds. Four distinct methods emerge in this domain: Mean, Median, Min, and Max consensus scoring. Each method, while unique in its approach, seeks to compute compound scores, harnessing quantile-normalized scores drawn from various docking programs. Yet, it is the introduction of advanced consensus strategies that truly exemplifies the study's innovation [18]. The mean–variance consensus and gradient boosting consensus stand out in this study, seamlessly merging advanced statistical models, gradient boosting mechanisms, and intricate algorithms to refine and enhance score computation [18]. With the debut of machine learning techniques, the introduction of the Deep Docking (DD) method marks the culmination of this research odyssey. This innovative method, fortified with the prowess of artificial intelligence, addresses the challenges posed by the exponential growth of chemical libraries, offering a beacon of hope for researchers navigating the intricate maze of virtual screening [19–21]. In our recent work, we introduced a workflow that combines four structure- and ligand-based scoring systems to improve the hit rate with a challenge of a narrow range of active compounds dataset. The results showed that the consensus scoring method outperformed separate screening methods, achieving the highest ROC value [22].

In this study, various protein targets, including G protein-coupled receptors (GPCRs), kinases, nuclear proteins, proteases, DNA repairing enzymes, and suppressor proteins, were explored. We introduce a novel consensus scoring method for holistic virtual screening. This method employs a sequence of machine learning models organized in a pipeline, with weights assigned based on individual model performance using a novel equation. We have developed an original formula, termed “W_new,” which integrates five coefficients of determination and error metrics into a single metric to assess model robustness. Using this pipeline, we comprehensively evaluated multiple molecular targets, scoring them based on docking, pharmacophore, shape similarity, and QSAR properties, which were used to train machine learning models. The selection of the optimal model, based on its assigned weight, enabled retrospective scoring of each dataset through a weighted average Z-score across the four screening methodologies. Additionally, we validated the robustness of these models using an external dataset to assess predictive performance and generalizability. Enrichment studies were conducted to evaluate the efficacy of the workflow.

## Methods

### Dataset

The datasets for this study were obtained from the PubChem database [23] and the Directory of Useful Decoys: Enhanced (DUD-E) repository [24], which were utilized to amass active compounds and corresponding decoys for the selected proteins. IC_50_ activity metrics were curated from PubChem, encompassing a range of forty to sixty-one active compounds per protein. Additionally, a substantial collection of decoys was meticulously compiled, numbering between 2300 and 5000 for each protein. To ensure the robustness and reliability of our study, an assessment for identifying and quantifying bias in datasets was conducted, addressing potential biases in active compound selection and decoy distribution. The active compounds were subsequently segregated into distinct sets for testing and validation, as well as for external validation purposes. The molecular structures were neutralized and compound duplication was removed, salt ions and small fragments were excluded. The IC_50_ values were further converted into pIC_50_ values using the formula pIC_50_ = 6 − log (IC_50_(μM)). Stereoisomers were systematically generated due to the presence of compounds characterized by undefined stereocenters within their SMILES representations.

### Assessment of datasets for identifying and quantifying bias

In this study, we employed a rigorous strategy to mitigate bias in analyzing active and decoy datasets for each target, bolstering the credibility of our findings. An essential aspect was the incorporation of an external validation dataset, unseen during model training. This, coupled with satisfactory R2 values, enhances the credibility of AUC and other performance metrics, confirming the robustness of our models. Additionally, our methodology deviates from conventional virtual screening practices, which typically maintain a 1:50 to 1:65 ratio of active to decoys [25–27]. By adopting a more stringent 1:125 ratio, we increase the challenge of accurately identifying actives within the decoy dataset. Notably, these performance metrics primarily facilitate comparative assessments between consensus scoring and other screening methods, demonstrating the superior efficacy and precision of consensus scoring.

In this assessment, we’ve employed a three-stage workflow to validate the datasets, following Sieg and Flachsenberg’s criteria for comparative analysis with MUV datasets to identify differences [28]. This methodology addresses issues highlighted by Sieg et al., particularly biases arising from uneven distributions of physicochemical properties among active and inactive groups, which can skew model outcomes. We also examined “analogue bias,” where numerous active analogues from the same chemotype inflate model accuracy. This approach enhances structural diversity within the datasets, reducing variability in predictive accuracy and yielding more robust and generalizable machine learning models [29].

We initially assessed seventeen physicochemical properties to ensure balanced representation between active compounds and decoys for each protein target. Fragment fingerprints were then used to prioritize diversity in compound selection and analyze patterns of similarity and diversity among active compounds and decoys. Two-dimensional principal component analysis (2D PCA) was applied to visualize the positioning of active compounds relative to decoys for each target. To refine the calculation of median active neighbors among decoys, adjustments were made to align with the actual decoy pool size and the 1:125 active-to-decoy ratio. This enhanced the evaluation of spatial relationships within chemical space and improved detection of compound distribution patterns and potential dataset biases. To compare with established datasets, we sampled two random datasets from the Maximum Unbiased Validation (MUV) dataset, maintaining the same active-to-decoy ratio used in our study [30, 31].

### Calculation of fingerprints and descriptors for active compounds and decoys

In this study, RDKit [32] open-source scripts were utilized to compute a wide range of molecular fingerprints and descriptors for both active and decoy compounds associated with each protein target. These descriptors encompassed Atom-pairs, Avalon, Extended Connectivity Fingerprints-4 (ECFP 4), (ECFP 6), MACCS, Topological Torsions fingerprints, as well as partial charges. Additionally, a set of ~ 211 descriptors provided by RDKit was incorporated as chemical compound features. For a comprehensive understanding of the specific features employed, the pertinent code snippets are available in the GitHub source repository.

### Selection of protein targets and crystal structures

We selected a carefully curated set of protein targets, including nuclear receptors, kinases, and enzymes, for investigation. These targets underwent robust validation using both active compounds and decoy ligands. Additionally, we deliberately excluded a subset of external datasets from the training and testing datasets to prevent data leakage and enable evaluation of the computational models’ predictive robustness. Crystal structures of macromolecular targets (AA2AR, AKT1, CDK2, DPP4, PPARG, and EGFR) were obtained from DUD-E, along with their corresponding sets of active and decoy ligands. Active compounds for TDP1 and the p53 suppressor protein were sourced from PubChem and the scientific literature, encompassing anthraquinones and chalcone chemical classes [33–35].

To prepare the protein and ligand structures for subsequent analyses, Autodock Tools were employed. Protein crystal structures were retrieved from the Protein Data Bank (PDB) [36], where hydrogen atoms were systematically added, and water molecules were effectively removed. Furthermore, the dimensions and resolution of the grid maps were established utilizing the AutoGrid tool. All compounds were subjected to docking against the reference receptor, confined within an 18 Å cubic enclosure centered around a co-cyrstalized ligand. Protonation states were computed for all proteins within a pH range of 7 ± 2, with the aim of aligning them with the physiological pH conditions. The redocking procedure was applied to all protein targets with their respective co-crystallized ligands.

### Pharmacophore scoring

In the analysis of each of the eight datasets, we conducted an assessment aimed at identifying the most diverse molecules, with the objective of quantifying their resemblance to the remaining compounds within the dataset. Utilizing the RDKit and SKlearn packages, an algorithm was employed to systematically traverse the data rows within the DataFrame. The ECFP4 for each compound were calculated, and these fingerprints were then subjected to K-means clustering using the scikit-learn KMeans algorithm. Notably, the selection of a cluster count within the range of three to five was made to ensure that each resultant cluster would distinctly represent a chemically disparate group. Each cluster was subjected to a superimposition process, enabling the detection of common pharmacophore attributes, guided by a set threshold mandating the minimum presence of 3 to 5 of these features. Pharmacophore features were computed for each cluster using phase module in Schrödinger suite [37]. Each compound was scored by the group of features calculated in its cluster. This module allowed us to generate a pharmacophore model that encapsulates the essential structural elements required for potent ligand binding. To assess the predictive power of our pharmacophore model, we calculated the Root Mean Square Error (RMSE) for each active compound based on their feature matches with the model. This quantitative measure provided a reliable indicator of the model's accuracy in predicting bioactivity.

### Docking scoring

The protein structures were retrieved in PDB format and processed using AutoDock Tools [7]. Active compounds were formatted accordingly and converted to PDBQT format using AutoDock Tools, which contains crucial ligand property information and is compatible with AutoDock. Ligand preparation involved adjustments for stereochemistry, protonation, and the addition of polar hydrogen atoms using AutoDockTools. Gasteiger partial charges were assigned, and details regarding rotatable bond torsions were incorporated into the PDBQT format. Identification of the protein's binding pocket was based on available structural data or by referencing the binding site with the co-crystallized ligand in the original PDB file. A cubic grid box was defined around this identified binding site, tailored to encompass the pocket adequately while allowing ample space for ligand exploration. Grid spacing was determined at an optimal value (0.375 Å) to balance computational efficiency and precision. Molecular docking involved exploring the optimal conformation and orientation of the ligand within the receptor's binding site. AutoDock Vina was utilized to accommodate flexible ligands, prioritizing conformations and binding interactions resembling those of the co-crystallized ligand to calculate docking scores.

### 2D fingerprint shape similarity scoring

From each of the eight datasets, we computed the most diverse molecules to evaluate their resemblance to the remaining compounds within the set. The code used RDKit and SKlearn to extract SMILES notations from the DataFrame, compute ECFP4 fingerprints, and perform K-means clustering with K-Means algorithm. To ensure each cluster had a representative compound, the number of clusters was limited to three or four. Representative compounds were determined by choosing those with the longest SMILES notation, ensuring greater complexity and diversity as a selection criterion [38]. Subsequently, shape similarities between each active compound and the reference compounds were computed using the Tanimoto similarity metric. This script serves to compare a specified chemical reference compound against a collection of additional compounds in a CSV file, quantifying their structural similarities via the Tanimoto coefficient. The highest index for each compound from the reference compound list was considered. Code snippets executed to perform this process have been added to the GitHub link.

### Development of the weighted metric (W_new) for evaluating machine learning models

A comprehensive ensemble of twelve machine learning models was employed, each offering adaptable parameter tuning through grid search techniques tailored to the specific requirements of each case. These models encompassed Decision Trees, K-Nearest Neighbors (KNN), AdaBoost, Random Forest, Linear Regression, Elastic Net Regression, Gradient Boosting, XGBoosting, and various Support Vector Regression (SVR) models, including linear, sigmoid, Radial Basis Function (RBF), and Nu-SVR kernels. These diverse models were seamlessly integrated into a unified codebase, offering two distinct options for feature selection: Principal Component Analysis (PCA) or Mutual Information (MI) feature selection. To assess the models' robustness and performance across different cases, we introduced a weighted ranking system based on five key evaluation metrics: R-squared (R^2^) for training and validation sets, Mean Squared Error (MSE), Root Mean Squared Error (RMSE), and Mean Absolute Error (MAE).

In the proposed composite metric formula, several statistical measures are integrated to comprehensively evaluate the performance of a model. The formula begins with the sum of squared R-values, R^2^_train + R^2^_val, which represents the proportion of the variance in the dependent variable that is predictable from the independent variables, so this sum reflects the total explanatory power of the model over both datasets. When both R^2^_train and R^2^_val are high, their sum, is also high. This sum is part of the numerator in the formula, so a higher sum of performance metrics (P) will contribute to a larger value of W_new [39].

1 $${P=R}_{train}^{2}+ {R}_{val}^{2}.$$

Additionally, the formula includes the sum of error metrics (E), namely MSE, RMSE, and MAE. This sum represents the aggregate magnitude of prediction errors, irrespective of their direction. These terms form the denominator in the main fraction of the formula. Lower values of MSE, RMSE, and MAE result in a smaller denominator. Since dividing by a smaller number results in a larger value, this will increase W_new [40].

2 $$E=MSE+RMSE+MAE.$$

We computed the absolute difference (D) between ${R}_{train}^{2}$ and ${R}_{val}^{2}$, and then create an adjustment factor to account for the discrepancy:

3 $$D= \left|{R}_{train}^{2}-{R}_{val}^{2}\right|.$$

Then, we added the adjustment factor to penalize discrepancies between training and validation performance:

4 $$A=\frac{1-D}{1+D}.$$

We combined the performance metric sum (P) with the error metric sum (E) and adjust based on the discrepancy adjustment factor (A):

5 $$W=\frac{P}{E}*A= \frac{{R}_{train}^{2}+ {R}_{val}^{2}}{MSE+RMSE+MAE}*\frac{\left(1-\left|{R}_{train}^{2}-{R}_{val}^{2}\right| \right)}{\left(1+\left|{R}_{train}^{2}-{R}_{val}^{2}\right|\right)}.$$

Finally, we normalized W to ensure it's within a specific range (0–1), by dividing it by $1+W$ [41, 42].

6 $$ W\_new =\frac{W}{1+W}.$$

Putting it all together, we get:

7 $${W\_new}= \frac{ \left(\left(\frac{\left({R}_{train}^{2} +{R}_{val}^{2}\right)}{\left(MSE + RMSE + MAE\right)}\right)*\frac{\left(1 -\left|\left({R}_{train}^{2} - {R}_{val}^{2}\right)\right|\right)}{\left(1 +\left|\left({R}_{train}^{2}-{R}_{val}^{2}\right)\right|\right)}\right)}{1+\left( \left(\frac{\left({R}_{train}^{2}+{R}_{val}^{2} \right)}{\left(MSE + RMSE + MAE\right)}\right)*\frac{\left(1 -\left|\left({R}_{train}^{2} -{R}_{val}^{2}\right)\right|\right)}{\left(1 +\left|\left({R}_{train}^{2}-{R}_{val}^{2}\right)\right|\right)}\right)}.$$

The proposed weight formula, “w_new,” assigns higher weights to models with superior performance, characterized by elevated R^2^ scores in training and validation, decreased MAE, RMSE, and MSE values, and smaller discrepancies between training and validation R^2^ scores, indicating resistance to overfitting. Conversely, models with lower performance receive reduced w_new values, as evidenced by diminished R^2^ scores, increased MAE, RMSE, and MSE values, and larger training-validation R^2^ score gaps. Notably, w_new is applicable when training and validation R^2^ scores range between 0 and 1, with the algorithm excluding results beyond this interval to ensure the identification of adequately performing models.

Furthermore, it's noteworthy that each individual machine learning model integrated into the aforementioned code was fine-tuned using the w_new formula. This fine-tuning process involved specific cross-validation techniques and the selection of an optimal number of PCA components or features through the script. This meticulous approach facilitated the identification of the best-performing machine learning model, characterized by the highest w_new value. All the code snippets, performed in this study, have been documented and made accessible on GitHub.

### Establishing predictive workflow through consensus holistic virtual screening

**Fig. 1:**
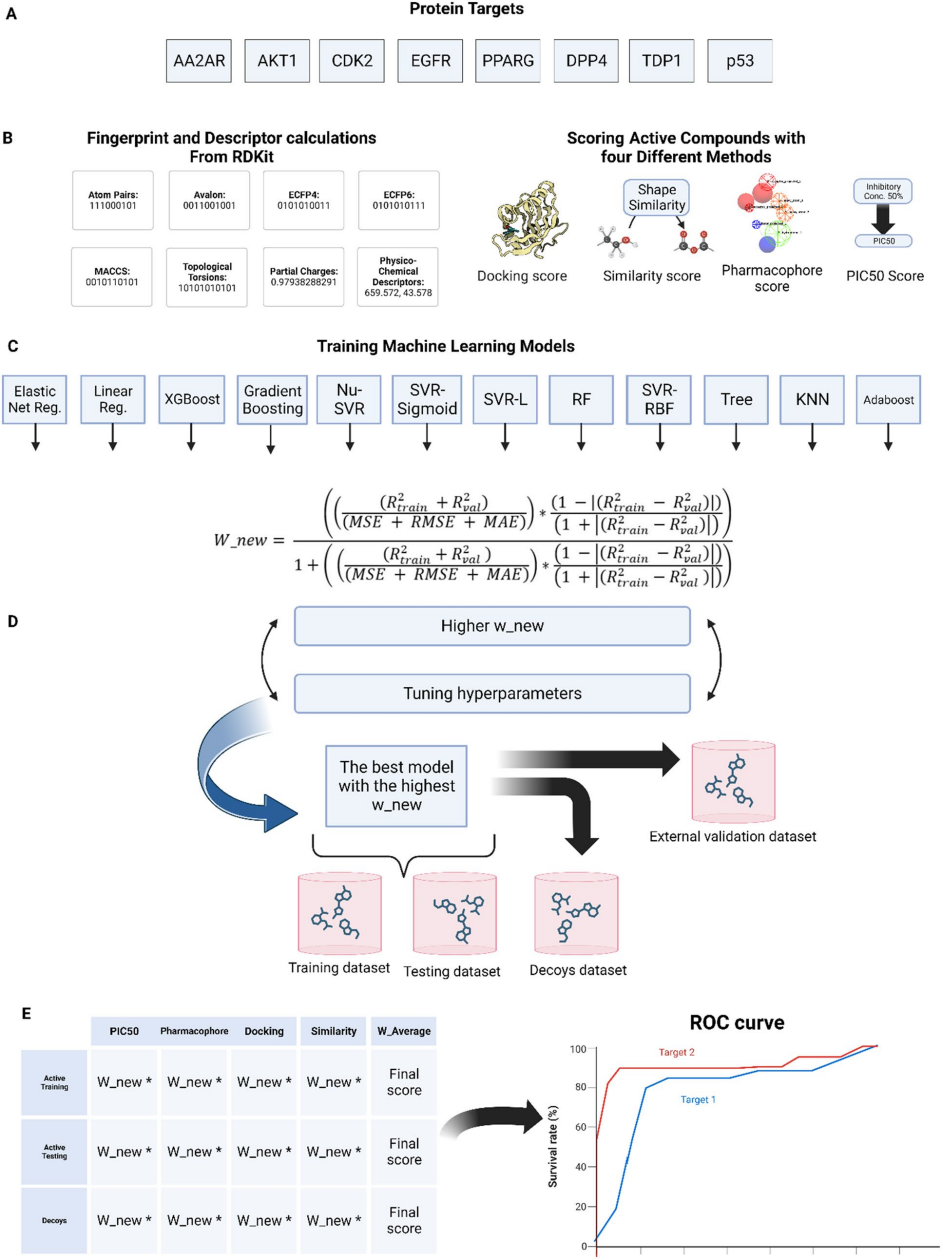
Comprehensive Workflow for the Consensus Holistic Virtual Screening. **A** Selection of protein targets spanning diverse categories, including G protein-coupled receptors (GPCR), kinases, nuclear proteins, proteases, and other targets. **B** Calculation of fingerprints and descriptors for both active and decoy datasets, along with the computation of four distinct scoring metrics for active datasets per target. **C** Integration of twelve machine learning models in the pipeline to identify the most optimal dataset within each scoring category. **D** Utilization of a novel formula to determine optimal parameters based on the highest w_new value. **E** Evaluation of the entire workflow's performance, including ROC curve analysis and other metrics, to demonstrate its effectiveness

Upon identifying the most robust model for each dataset using four scoring methods—PIC50 (QSAR), pharmacophore, docking, and shape similarity—a detailed evaluation was conducted. This included training each model on a training dataset and evaluating its performance on a separate validation dataset, split in a 70:30 ratio. Further validation was performed on an external dataset to compute R^2^ and confirm prediction robustness. For a holistic model assessment, both active and decoy compounds were scored using the same approach, with scores standardized via z-scoring. Each score was then adjusted by the w_new factor from the previous step. A weighted average score was calculated for each compound, leading to their descending order ranking based on these scores. This ranked list underpinned the creation of an enrichment curve, as depicted in Fig. 1.

## Results

In this study, we analyzed eight protein targets across diverse functional categories, including GPCRs, kinases, nuclear proteins, proteases, DNA repair enzymes, and tumor suppressor proteins. Table 1 details the meticulous examination of active and decoy compounds sourced from the DUD-E database for each target. Notably, active compounds for TDP1 and p53 were exclusively selected from anthraquinone and chalcone chemical classes, sourced from PubChem, BindingDB, and literature. Decoy sets for these targets were generated using the “Generate Decoys Tab” in DUD-E. This departure aimed to evaluate the efficacy of the consensus holistic virtual screening strategy across diverse datasets. Additionally, the methodology was evaluated for its impact on performance metrics within new settings [32], building on previous evaluations. External datasets were used for predictive capability assessment, and R2 values were calculated for validation.

**Table 1:** Enumeration of the protein targets studied, detailing counts of active compounds, decoys, the number of external validation datasets, and the respective PDB IDs used to score active compounds within each target dataset

Protein target	No. of actives	No. of decoys	No. of actives in the external validation	PDB ID
AA2AR	40	5000	10	3EML
AKT1	40	5000	10	3CQW
CDK2	40	5000	10	1H00
DPP4	47	5000	10	2I78
TDP1	51	2700	11	6N0D
PPARG	43	5000	10	2GTK
EGFR	50	5000	10	2RGP
P53	20	2300	5	6GGB

### Comparative analysis of bias in datasets distribution and diversity

**Fig. 2:**
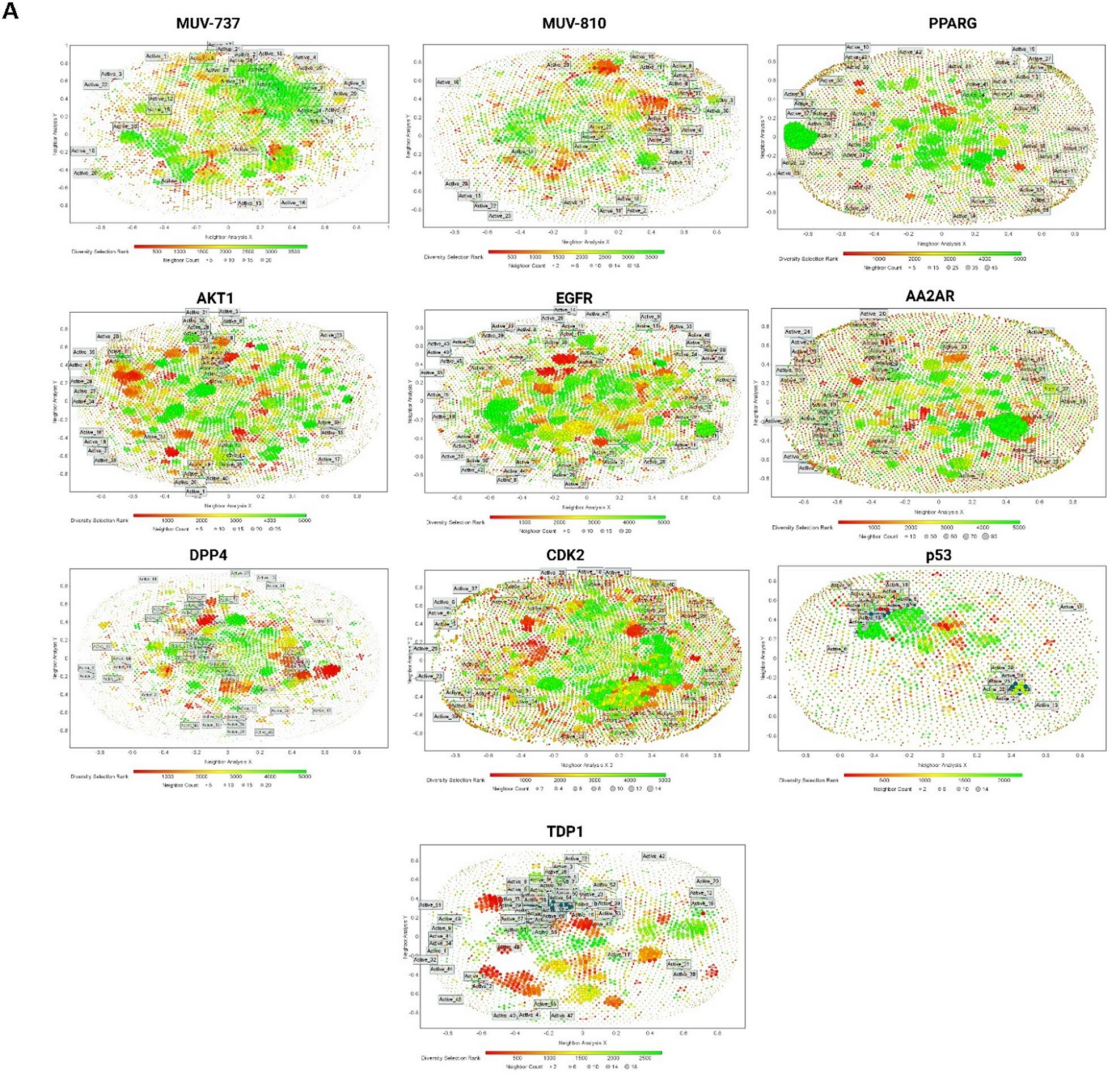

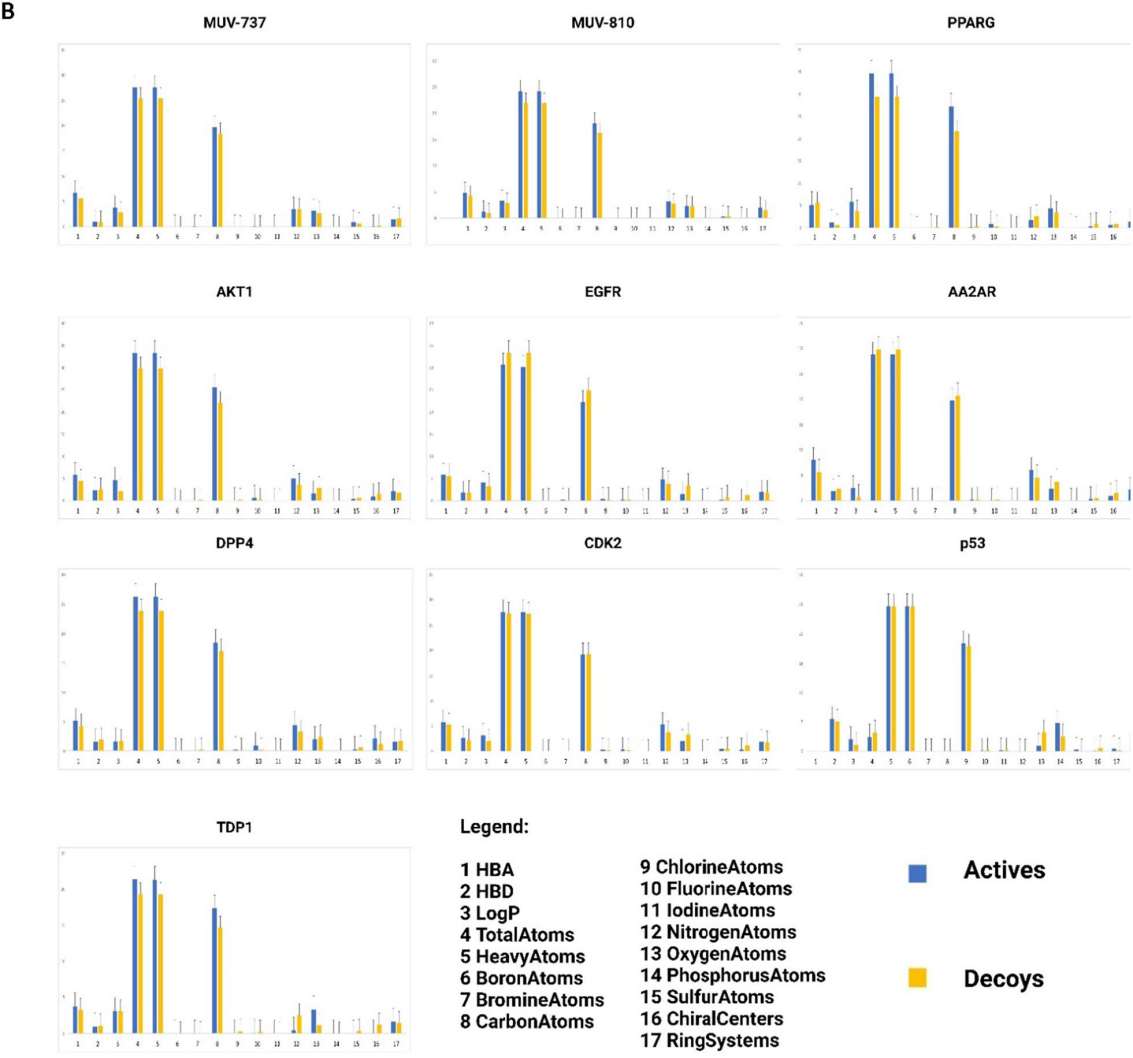

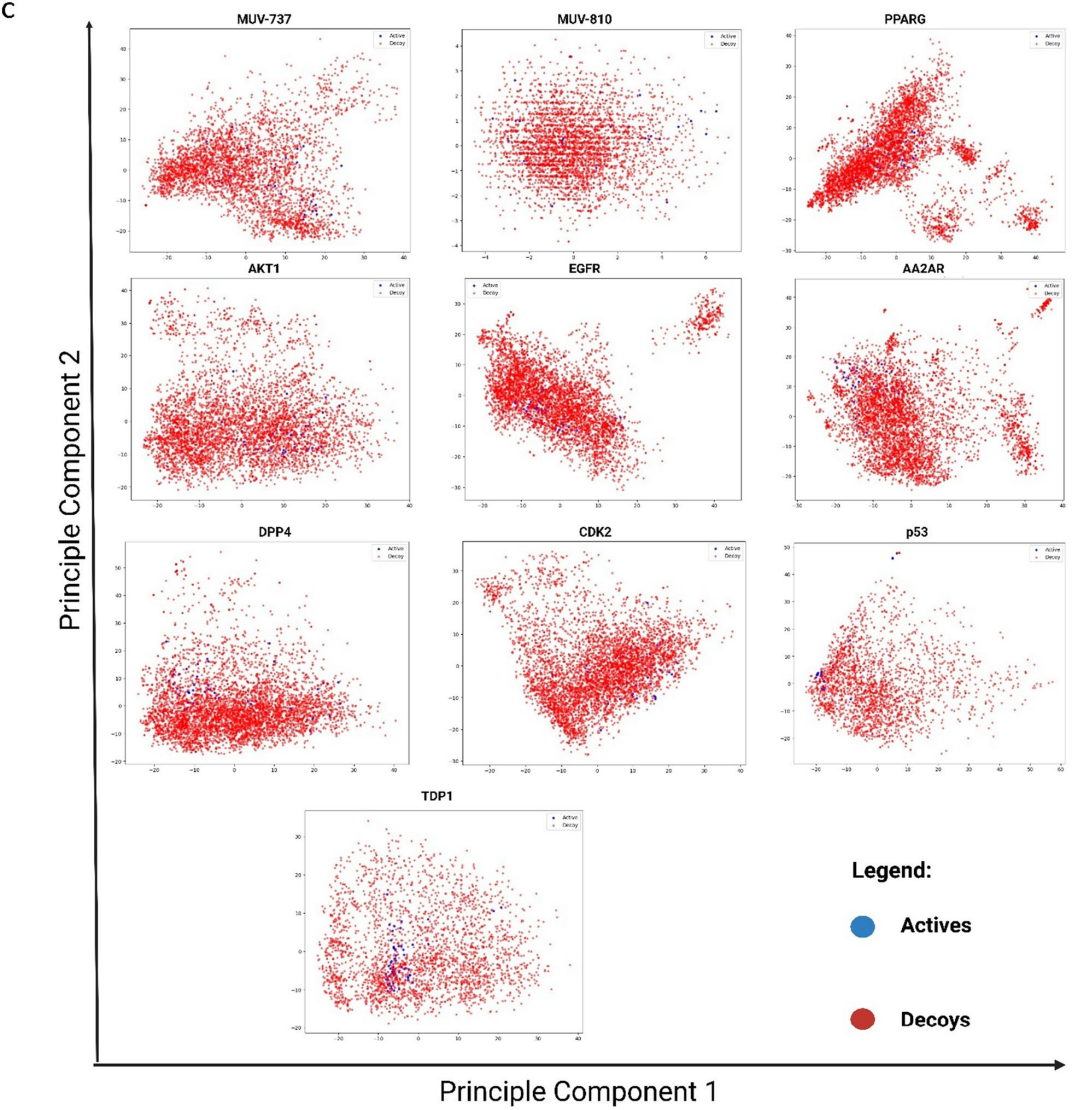
Comparative analysis of active compounds and decoys across eight datasets employed in this study and two MUV datasets. **A** Similarity maps, generated via the 2D Rubber Band Scaling algorithm utilizing fragment fingerprints, depict the spatial arrangement of active compounds in comparison to decoys. These maps are color-coded according to the diversity selection rank, offering a visual representation of the compounds' distribution. **B** Distribution of physicochemical properties for seventeen distinct properties between actives and decoys. **C** Principal Component Analysis maps constructed from eight types of descriptors, demonstrating the segregation of active compounds from decoys

Figure 2A displays the distribution of active compounds among decoys across each target protein, along with their neighboring active and decoy compounds. Except for TDP1 and p53, distribution patterns across other targets closely resembled those in MUV datasets (particularly MUV-737 and MUV-810). Active compounds were positioned in central and peripheral regions, indicating diverse interactions with other actives and decoys. Deviation in TDP1 and p53 datasets is attributed to their unique composition with anthraquinone and chalcone derivatives, suggesting stronger connections among themselves and differentiation from decoys. These datasets were designed to explore dataset incompatibilities, as previously studied [22], and their influence on performance metrics was assessed in the current study.

The Rubber Band Scaling algorithm used in the similarity maps assigns compounds random positions in a quadratic space to minimize the distance between them. Optimization cycles adjust compound positions based on their similarity relationships defined by the Fragment Fingerprint descriptor. Compounds are moved closer or further apart to reflect their chemical similarity, ensuring similar compounds are close neighbors in the visualization [43]. The maps are color-coded based on diversity selection ranking, with higher values indicating less diversity (green) and lower values indicating more diversity (red). Similarity in this metric between active compounds and decoys suggests homogeneity in chemical class diversity. However, greater diversity among active compounds can enhance heterogeneity in training and testing sets, minimizing bias in machine learning scoring functions, as described by Li and Yang [29]. Refer to the Supplementary Material 3 file for a detailed view of the components in Figure 2A, B, and C.

Data from the similarity maps, presented in Table 2, reveal average diversity rank differences between active compounds and decoys across various target datasets. The diversity range of these datasets aligns with that of the two MUV datasets in this study, facilitating comparative diversity analysis against a recognized benchmark. Notably, some datasets, like DPP4, show no significant diversity differences between actives and decoys, while most exhibit significant differences. Unlike MUV-810 and DPP4, most datasets feature more diverse actives (lower values) than decoys, potentially enhancing training and testing compound diversity relative to decoys [44]. The most pronounced differences in diversity ranks between active compounds and decoys were identified within the TDP1 and p53 datasets, translating the graphical clustering of active compounds into a quantifiable disparity in diversity rank. This distinction does not imply higher overall diversity but rather delineates the active compounds' separation from decoys, attributed to their aggregation in confined areas of the maps.

**Table 2:** Analysis of the mean diversity rank of active compounds and decoys, significant and insignificant differences between actives and decoys in the physicochemical properties, and the corrected median number of neighbors for actives within the PCA framework in the dataset targets

Dataset	Actives mean diversity rank	Decoys mean diversity rank	Physicochemical Significant Differences		Number of median nieghbors for the actives in the PCA	Corrected number of median nieghbors for the actives in the PCA
			Significant	Non-Significant		
AA2AR	1851.72^*^	2525.85	7	10	444.50	444.50
AKT1	1717.10^*^	2526.92	8	9	563.00	563.00
CDK2	1783.22^*^	2526.39	6	11	395.00	395.00
DPP4	2651.82	2522.29	9	8	576.00	490.21
TDP1	448.11^*^	1402.07	10	7	167.00	112.23
PPARG	1798.53^*^	2527.71	10	7	567.00	527.44
EGFR	1471.02^*^	2546.04	10	7	371.50	371.50
P53	577.25^*^	1132.39	9	8	74.00	74.35
MUV-737	2277.36^*^	1887.40	6	11	415.50	552.60
MUV-810	2210.06	1887.15	7	10	239.00	318.60

*Indicates a significant difference between actives and decoys in the T-test at p < 0.05

In Fig. 2B, seventeen physicochemical properties were computed for all datasets and compared with two MUV datasets. The minimal differences between actives and decoys across the protein target datasets, ranging from 7 to 11, mirror the consistency seen in the MUV datasets, where 10 to 11 non-significant property differences were observed in MUV-810 and MUV-737, respectively (see Table 2). This indicates fewer disparities between actives and decoys, enhancing dataset reliability and comparability with established benchmarks [45]. In the final validation phase, PCA was used to visualize both active compounds and decoys, incorporating all utilized fingerprints and descriptors from model training. Classification was performed to differentiate between active and decoy compounds based on predefined titles, enabling focused examination of molecular characteristics distinguishing active compounds from inactive ones. Euclidean distances between each active compound and all decoys within the dimensionally reduced space were computed, with a threshold distance set by the 10th percentile of these distances facilitating identification and enumeration of decoys considered 'neighbors' to each active compound. This neighbor count served as an indicator for assessing the similarity level between actives and the decoy-dominant chemical space. The analytical results were summarized into a statistical metric, the median number of neighbors, subsequently normalized against the decoy count and the active-to-decoy ratio percentage. For a graphical representation of this process, refer to Fig. 2C.

As demonstrated in Table 2, AKT1 exhibited the highest median number of active neighbors among decoys, with a value of 563, followed by MUV-737 at 552.6. Conversely, TDP1 and p53 displayed the lowest median numbers of neighbors, at 112.23 and 74.35, respectively, with MUV-810 showing the third lowest at 318.60. The diminished neighbor count observed for these active compounds suggests a higher selectivity or a lower chemical similarity compared to actives surrounded by a greater number of decoy neighbors [46]. As previously mentioned, the actives within the TDP1 and p53 datasets belong to two distinct chemical classes, leading to a propensity for clustering amongst themselves rather than mingling with decoys. This distribution highlights how the protein datasets in question align with the benchmark established by the MUV dataset.

### Analysis of different screening scores across macromolecular targets

**Fig. 3:**
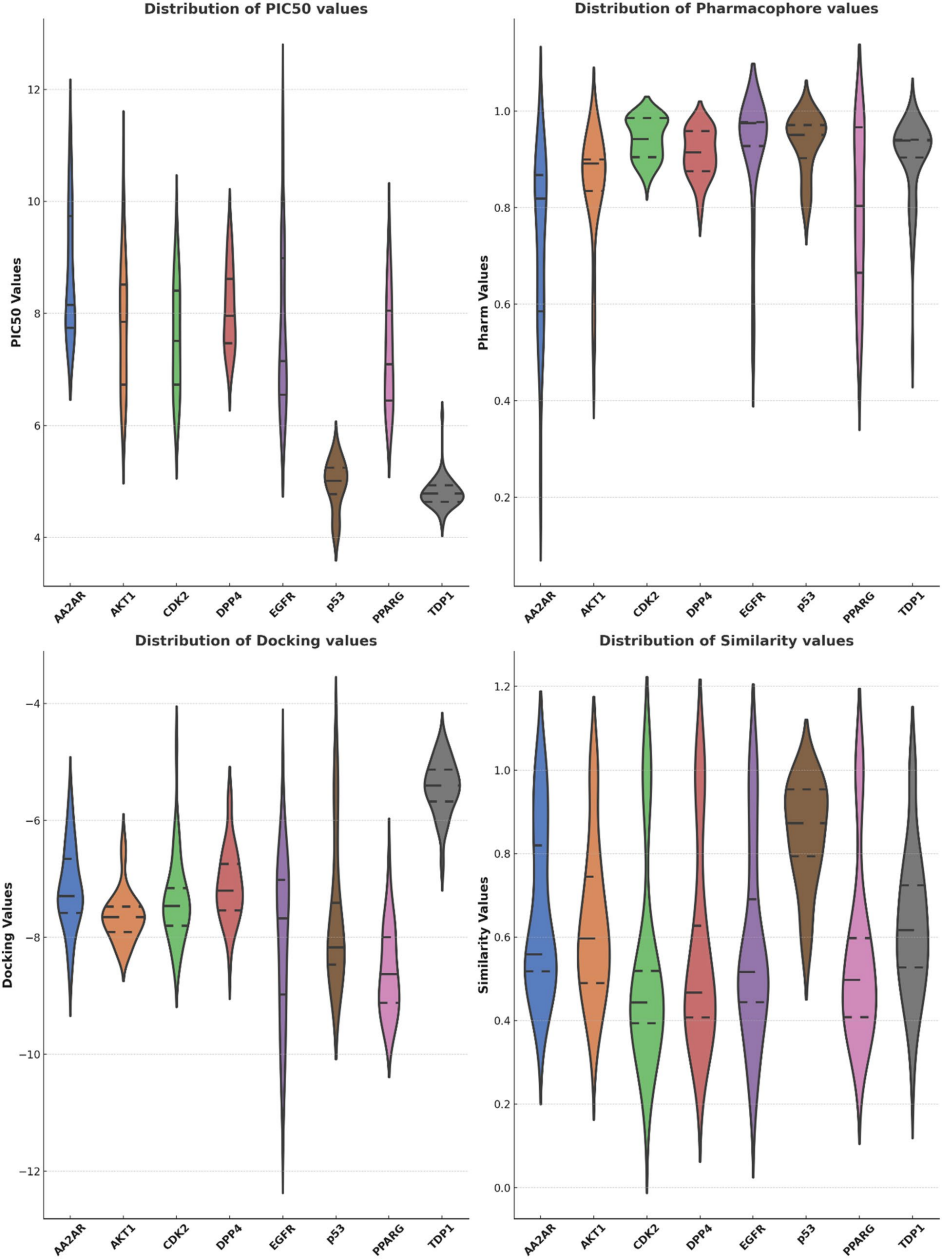
Distribution of various bioactivity metrics across different protein targets. The four panels represent the distributions of Docking, PIC50, Pharmacophore, and Similarity values for eight protein targets (AA2AR, AKT1, CDK2, DPP4, TDP1, PPARG, EGFR, and p53). Each violin plot depicts the distribution of values for the respective metric, with the width of the plot at different values indicating the density of data points. The inner lines represent quartiles of the distribution

It is noteworthy, as illustrated in Fig. 3, that the distribution of PIC_50_ values of both p53 and TDP1 diverges significantly from the broader spectrum of other macromolecular targets, with the latter target dataset exhibiting a considerably wider range of activities. Additionally, we must highlight the relatively balanced distribution observed across various scoring metrics, encompassing pharmacophore analysis, docking simulations, and similarity scoring for both TDP1 and p53. Of particular interest is the exceptional performance observed in the case of similarity scores, which are distributed more evenly across the entire cohort of targets. In contrast, pharmacophore scores, followed by docking scores, reveal less uniform distributions for specific targets. Nevertheless, it becomes apparent that distinct computational methodologies yield varying levels of performance, not intrinsically associated with their respective average “w_new” values. Among these methodologies, the pharmacophore approach emerges as the most robust, displaying the highest average “w_new” value of ~ 0.965. Closely following, the shape similarity method demonstrates commendable performance, with an average “w_new” value of ~ 0.895. Conversely, the results of Docking screening yield a comparatively lower average “w_new” value, ~ 0.681. Lastly, the “PIC_50_” scoring approach exhibits the least favorable performance, denoted by its lowest average “w_new” value of ~ 0.671. These findings underscore the considerable variability in the predictive capabilities of these screening methodologies within the context of our study.

### Machine learning models generated and their performance

**Fig. 4:**
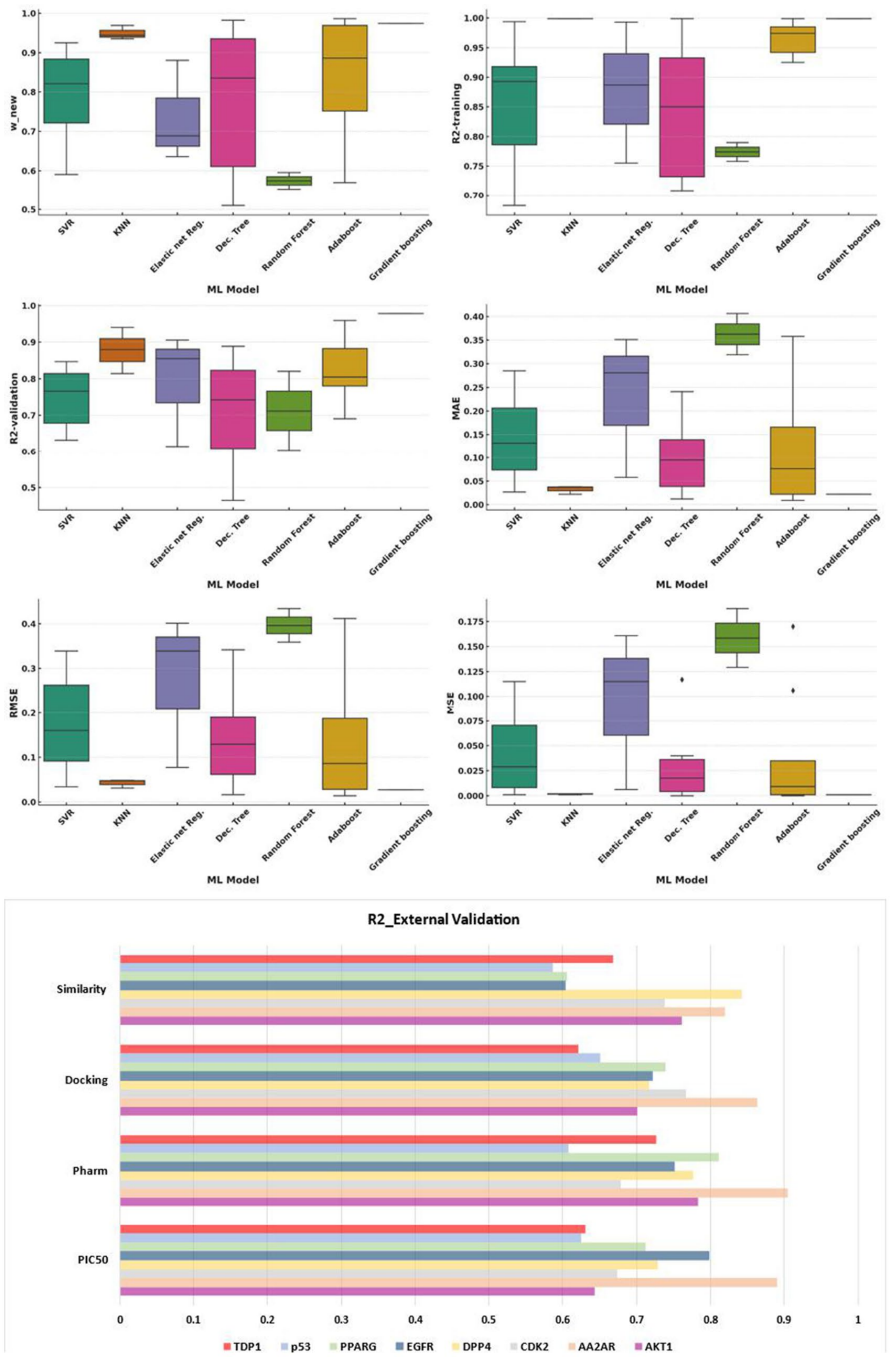
Comparative Analysis of Machine Learning Model Performances in the consensus holistic workflow: The upper panel presents a series of box plots showcasing the distribution of performance metrics such as R^2^ validation and training, W_new, MAE, MSE, and RMSE for various machine learning models. The lower panel illustrates the R^2^ values for external validation of four key predictive features—PIC_50_, Pharm, Docking, and Similarity—across multiple target proteins, providing insights into the predictive accuracy and reliability of the models employed

In this study, several machine learning models exhibit distinct performance metrics. SVR models that include all kinds of SVR and Nu-SVR models with different kernels, on average, yield an R^2^-training score of ~ 0.854 and an R^2^-validation score of 0.749. Its MAE stands at 0.147, with an RMSE of about 0.180. The Adaboost models achieve an average R^2^-training score of 0.967 and an R^2^-validation score of 0.825. Decision Trees, characterized by a more flexible structure, report an R^2^-training value of 0.843 and an R^2^-validation value of 0.709. The Elastic Net and linear Regression models present an R^2^-training score of 0.878 and a validation score of 0.792. Gradient Boosting, a boosting ensemble method widely used in QSAR modeling [47], showcases impressive scores with an R^2^-training of 0.999 and an R^2^-validation of 0.978. The k-Nearest Neighbors (KNN) models register an R^2^-training score of 0.999 and a validation score of 0.878. Across these models, the w_new parameter displays a range of values, with Gradient Boosting exhibiting the highest average value of 0.974, suggesting its superior performance in the given context as depicted in Fig. 4.

The evaluation of an external validation dataset reveals variable predictability among proteins, with R^2^ values ranging from 0.625 for p53 to 0.891 for AA2AR, reflecting differences in inhibitory concentrations. High R^2^ values for AA2AR (0.891) and EGFR (0.797) indicate potent inhibitory effects, demonstrating the models' predictive accuracy. Pharmacophore scores, particularly for AA2AR (R^2^ = 0.905) and PPARG (R^2^ = 0.810), suggest reliable pharmacophore model predictions. Docking scores vary, with CDK2 (R^2^ = 0.766) and PPARG (R^2^ = 0.739) indicating precise docking efficiency predictions. The analysis of 2D fingerprint shape similarity metrics shows significant variation, with DPP4 and TDP1 exhibiting higher scores, while p53's lower value is attributed to the dataset's small size, as shown in Fig. 4.

In the pursuit of robust scoring methods for producing a robust consensus holistic virtual screening within a diverse set of molecular targets, various machine learning models and kernels were employed, each yielding specific w_new values indicative of their performance. The Docking scoring method primarily employed SVR ML models with an RBF kernel, resulting in a w_new value of 0.872. In contrast, the QSAR (PIC_50_) scoring method utilized the same SVR ML model with an RBF kernel, yielding w_new average value of 0.888. The shape similarity scoring method was predominantly associated with the Adaboost ML model, which produced w_new value at 0.969. Similarly, the pharmacophore scoring method was best represented by the Adaboost ML model, achieving the highest w_new value of 0.986 among all scoring methods screened as illustrated in Table 3.

**Table 3:** Machine learning models used for each target protein within four screening methods, PIC_50_, pharmacophore, docking, and shape similarity with models’ performance metrics

Target protein	Scoring method	ML model	Model metrics						
			R^2^-train	R^2^-val	MAE	RMSE	MSE	w_new	R^2^-ext
AA2AR	PIC_50_	SVR RBF	0.910	0.838	0.270	0.339	0.115	0.673	0.891
	pharm	KNN	0.999	0.880	0.037	0.048	0.002	0.944	0.905
	Docking	Elastic net Reg	0.887	0.855	0.281	0.339	0.115	0.689	0.864
	Similarity	Nu-SVR linear	0.943	0.789	0.073	0.089	0.008	0.882	0.818
TDP1	PIC_50_	Dec. Tree	0.999	0.465	0.125	0.200	0.040	0.549	0.631
	pharm	Dec. Tree	0.914	0.889	0.030	0.049	0.002	0.955	0.726
	Docking	Dec. Tree	0.939	0.586	0.241	0.342	0.117	0.510	0.621
	Similarity	Elastic net Reg	0.755	0.614	0.058	0.077	0.006	0.880	0.667
EGFR	PIC_50_	Random forest	0.790	0.820	0.406	0.434	0.188	0.596	0.797
	pharm	Adaboost	0.925	0.786	0.009	0.014	0	0.983	0.791
	Docking	Adaboost	0.992	0.883	0.340	0.403	0.106	0.625	0.721
	Similarity	Adaboost	0.999	0.863	0.067	0.086	0.007	0.899	0.603
Akt1	PIC_50_	SVR RBF	0.682	0.743	0.185	0.216	0.047	0.738	0.642
	pharm	KNN	0.999	0.940	0.022	0.031	0.001	0.969	0.782
	Docking	SVR RBF	0.812	0.806	0.176	0.242	0.059	0.770	0.700
	Similarity	Adaboost	0.974	0.805	0.076	0.076	0.011	0.886	0.761
DPP4	PIC_50_	Nu-SVR RBF	0.883	0.847	0.088	0.104	0.011	0.888	0.728
	pharm	Nu-SVR RBF	0.994	0.632	0.027	0.034	0.001	0.925	0.776
	Docking	Adaboost	0.942	0.760	0.166	0.187	0.035	0.752	0.716
	Similarity	Adaboost	0.979	0.901	0.022	0.028	0.001	0.969	0.842
CDK2	PIC_50_	Random Forest	0.758	0.604	0.319	0.359	0.129	0.553	0.674
	pharm	Dec. Tree	0.786	0.809	0.012	0.016	0	0.982	0.678
	Docking	SVR RBF	0.708	0.644	0.074	0.092	0.008	0.872	0.766
	Similarity	Dec. Tree	0.708	0.828	0.064	0.099	0.010	0.874	0.737
PPARG	PIC_50_	Adaboost	0.939	0.780	0.358	0.412	0.170	0.570	0.711
	pharm	Gradient boosting	0.999	0.978	0.022	0.027	0.001	0.974	0.810
	Docking	Nu-SVR RBF	0.903	0.690	0.285	0.324	0.105	0.591	0.739
	Similarity	KNN	0.999	0.814	0.038	0.047	0.002	0.935	0.605
P53	PIC_50_	Dec. Tree	0.714	0.676	0.144	0.159	0.025	0.797	0.624
	pharm	Adaboost	0.985	0.959	0.012	0.015	0	0.986	0.607
	Docking	Elastic net Reg	0.993	0.906	0.351	0.401	0.161	0.636	0.651
	Similarity	Adaboost	0.969	0.691	0.086	0.092	0.009	0.834	0.586

### Factors influencing w_new values

**Fig. 5:**
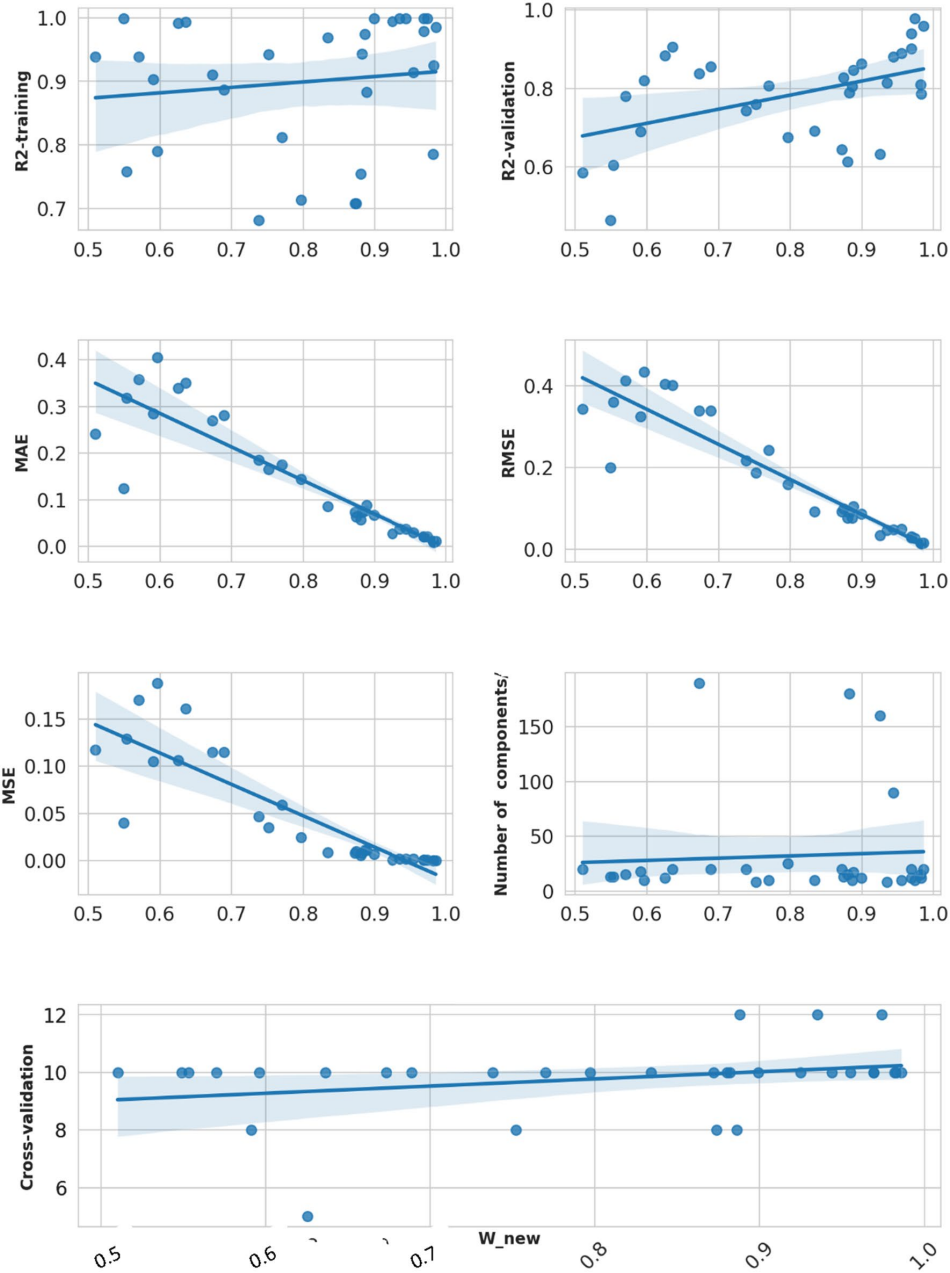
Pairplot shows the correlations between performance metrics and models parameters, cross-validation, and numbers of PCA and features components

To find out the factors with a higher influence on w_new and the effects of model complexity against performance metrics we employed several techniques. We analyzed the correlation between the five performance metrics previously mentioned with cross-validation times, number of PCA components/features, and model parameters such as model cost and gamma, Nu value in SVR, L1 (Lasso), and L2 (Ridge) regularization in addition to other hyperparameters according in the model employed as clarified in the Supplementary information Table 1. From Fig. 5, The correlation coefficients between w_new and the various metrics are as follows: R^2^-training equals 0.1265, R2-validation is 0.4638, MAE =  − 0.9022, RMSE =  − 0.9324, MSE =  − 0.8729. R^2^-training and R^2^-validation have positive mild and moderate correlations with w_new, respectively. However, the correlation with MAE, RMSE, and MSE have strong negative correlations with w_new. As the error metrics increase, w_new tends to decrease. Among the error metrics, RMSE has the strongest negative relationship with w_new, followed by MSE and then MAE.

In pursuit of a deeper understanding of the contributions of various metrics to the variable w_new, a multiple linear regression analysis was conducted. This rigorous examination sought to discern the individual influence of each metric on w_new while effectively controlling for the presence of other metrics. The formulated multiple linear regression model is articulated as follows:

$${w}_{\_new} = \beta 0 +\beta 1\times {R}_{train}^{2}+\beta 2\times {R}_{validation}^{2}+\beta 3\times MAE+\beta 4\times RMSE+\beta 5\times MSE+\epsilon .$$

The multiple linear regression model constructed here consists of β0 representing the intercept, while β1 to β5 correspond to the coefficients of the variables, and ϵ denotes the error term. Analysis of these coefficients reveals the relationship between w_new and the metrics as follows: β0, the intercept, at 0.7902 indicates the predicted value of w_new when all variables are at zero. β1 (R^2^-training) suggests a decrement of 0.1588 in w_new per unit increase in R^2^-training, holding other variables constant. Conversely, β2 (R^2^-validation) shows an increase of 0.4065 in w_new per unit rise in R^2^-validation, with other variables fixed. β3 (MAE) implies w_new increases by 0.6306 for each unit escalation in MAE, controlling for other variables. β4 (RMSE) indicates a reduction of 1.5866 in w_new per unit augmentation in RMSE, maintaining other variables. β5 (MSE) reveals an increase of 0.1938 in w_new for each unit increase in MSE, with other variables steady. The error term (ϵ) coefficient demonstrates a marginal positive influence on w_new, quantified at 0.0002. Statistical significance was assessed using associated p-values, where p-values < 0.05 were considered significant. The analysis indicates significant coefficients for R^2^-training, R^2^-validation, RMSE, and the error term, while MAE and MSE may not be statistically significant predictors of w_new when considered alongside other variables. Overall, R^2^-validation and RMSE emerge as the most influential factors impacting w_new, based on their coefficient magnitudes and statistical significance levels. These findings suggest that factors such as PCA/features components, parameters of each model, and cross-validation times have less impact on w_new.

### The effects of different factors on w_new in individual models

The exploration of various machine learning models unveiled consistent patterns in the relationship between the parameter w_new and model performance metrics. Across models like Adaboost, Decision Tree, Elastic Net Regression, SVR, and KNN, w_new displayed discernible associations. Notably, positive correlations were observed between w_new and certain performance indicators like 'Cross-validation' and R^2^-validation, suggesting that higher w_new values align with improved validation scores. Conversely, w_new consistently exhibited negative relationships with error metrics such as RMSE, MAE, and MSE, indicating that an increase in w_new corresponded to decreased error rates across models. Additionally, some models showcased nuanced relationships between w_new and specific parameters, like 'Minimum sample split' in the Decision Tree and 'Model gamma' in SVR. Overall, the consistent trends suggest that w_new plays a significant role in influencing model performance, particularly in relation to validation scores and error metrics, across diverse machine learning models [48]. See the Supplementary Fig. 1 for more details.

### The effects of hyperparameters on w_new in individual models

**Fig. 6:**
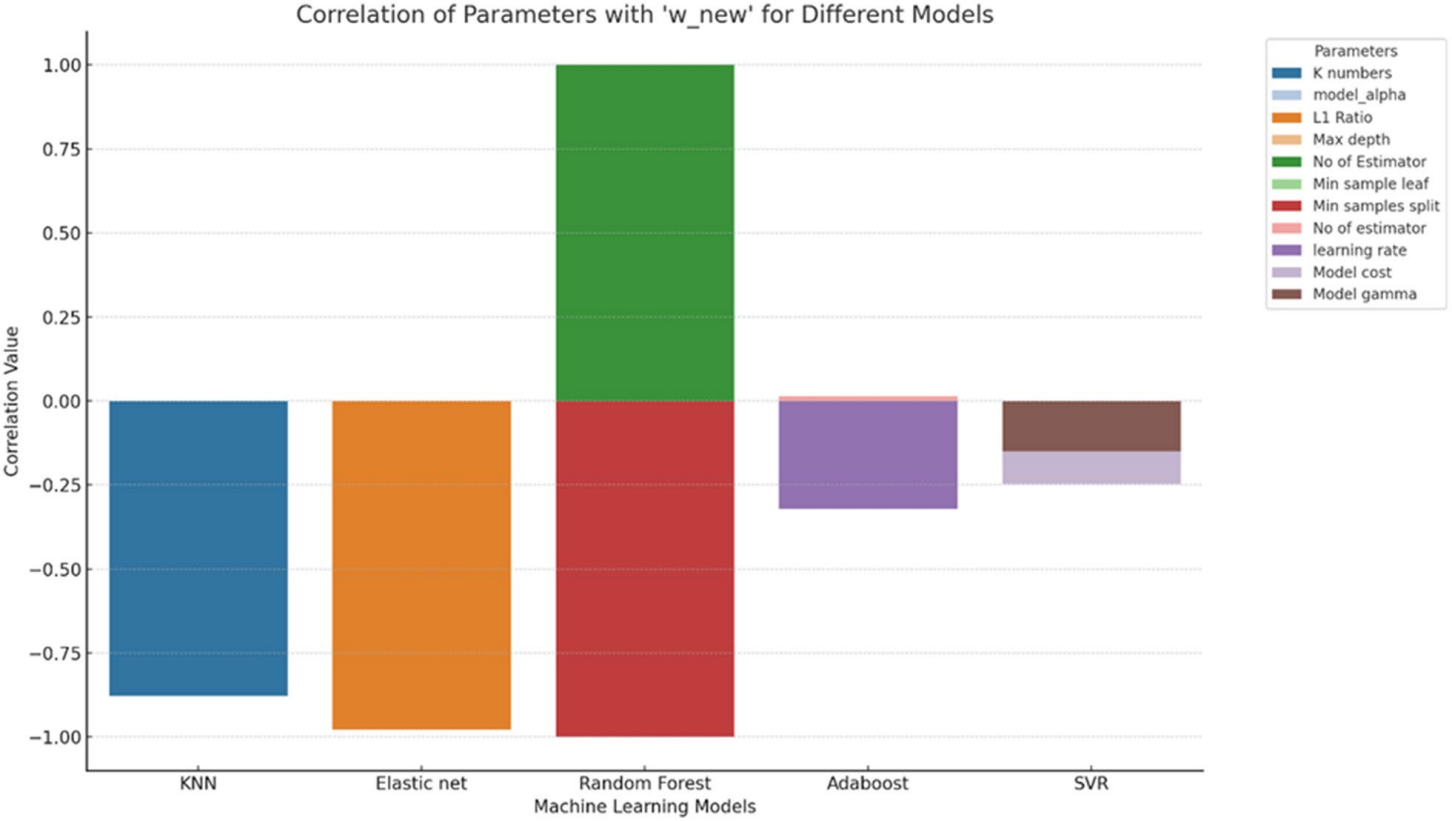
Bar plot illustrating the correlation strengths between various model parameters and the metric ‘w_new’ across different machine learning models. Each bar represents the correlation value of a specific parameter with ‘w_new’ for a given model. Positive values indicate a direct relationship, while negative values suggest an inverse relationship between the parameter and ‘w_new’

In computational modeling, the relationship between model complexity and hyperparameters, particularly in KNN models, highlights the critical influence of the number of neighbors (“K”) on model performance, showing a negative correlation of -0.877 with w_new. Decreasing “K” simplifies the model and improves prediction accuracy, notably in shape similarity and pharmacophore models, diverging from other QSAR model outcomes [49]. For Elastic Net models, model_alpha and the “L1 Ratio” hyperparameters significantly impact complexity, with negative correlations of − 0.349 and − 0.978 with w_new, respectively, indicating their strong influence on reducing model complexity [50]. Refer to Fig. 6 for a visualization of these relationships.

The Random Forest model demonstrates complexity modulation through hyperparameters, where “Max depth” and “Number of Estimators” exhibit high positive correlations with w_new, indicating an increase in model intricacy as these parameters increase [51] as depicted in Fig. 6. Conversely, “Min sample leaf” and “Min samples split” show significantly high negative correlations with w_new, implying a decrease in w_new with the escalation of these parameters [52]. In the Adaboost models, the “Number of Estimators” shows a slight positive correlation (0.014) with w_new, while the “learning rate” exhibits a significant negative correlation (− 0.321), suggesting a decrease in model complexity with a higher learning rate. In SVR, the “Model cost” and “Model gamma” parameters show negative correlations of − 0.247 and − 0.149 with w_new, respectively, indicating their roles in slightly reducing model complexity as they increase [53, 54].

Overall, the analysis highlights the varied impacts of hyperparameters on model complexity, with some leading to increased complexity and others to simplification, depending on the model and hyperparameter [55]. Simplified models favored in this study enhance interpretability and computational efficiency, offering advantages in real-time scenarios and environments with limited computing capacity [56]. Moreover, their simplicity is advantageous in situations with restricted data availability, showcasing superior performance relative to more complex models prone to overfitting and sensitivity to noise in sparse datasets [57].

### Enrichment metrics for the consensus holistic scoring in comparison to individual screening methods

**Fig. 7:**
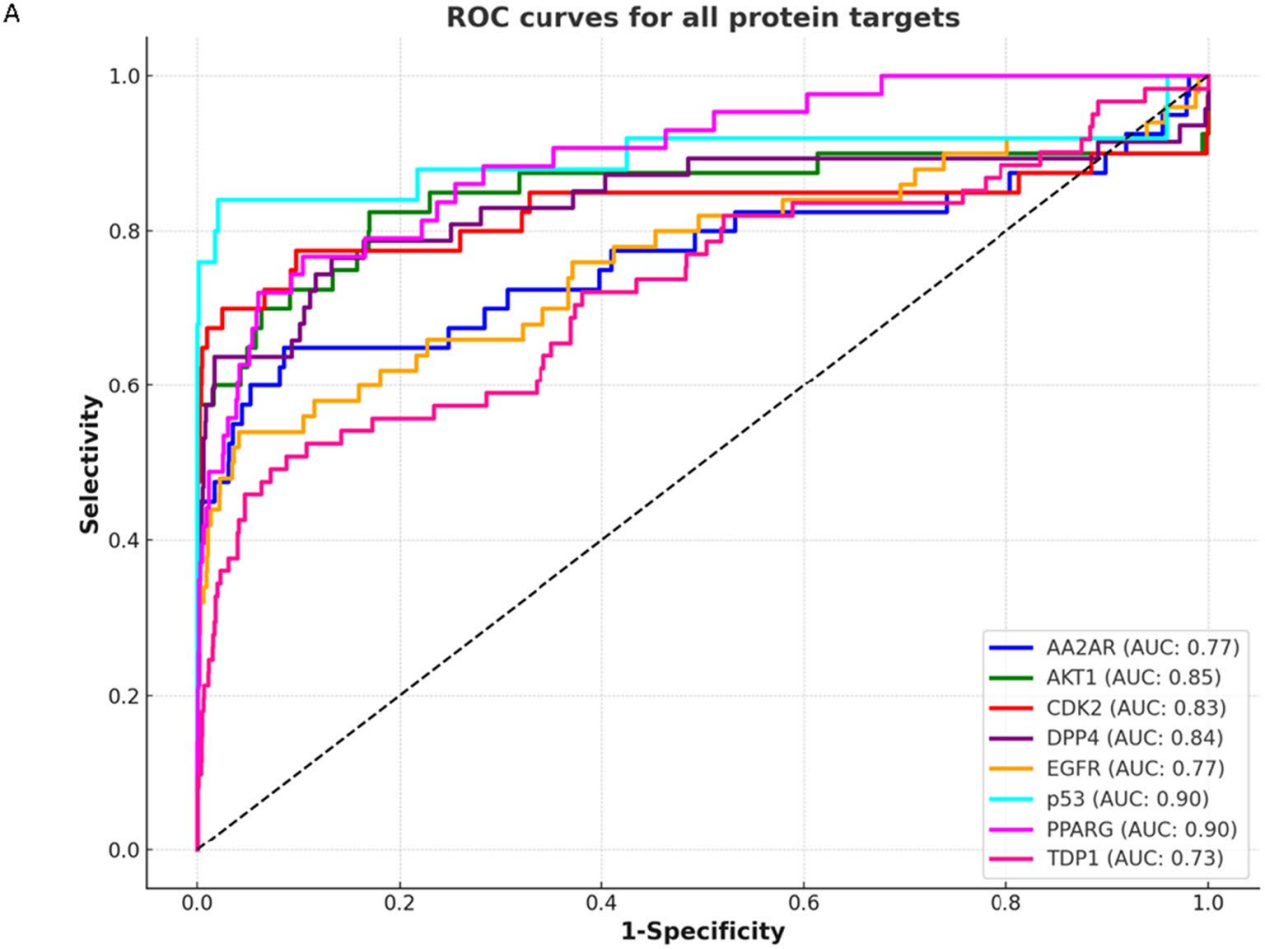

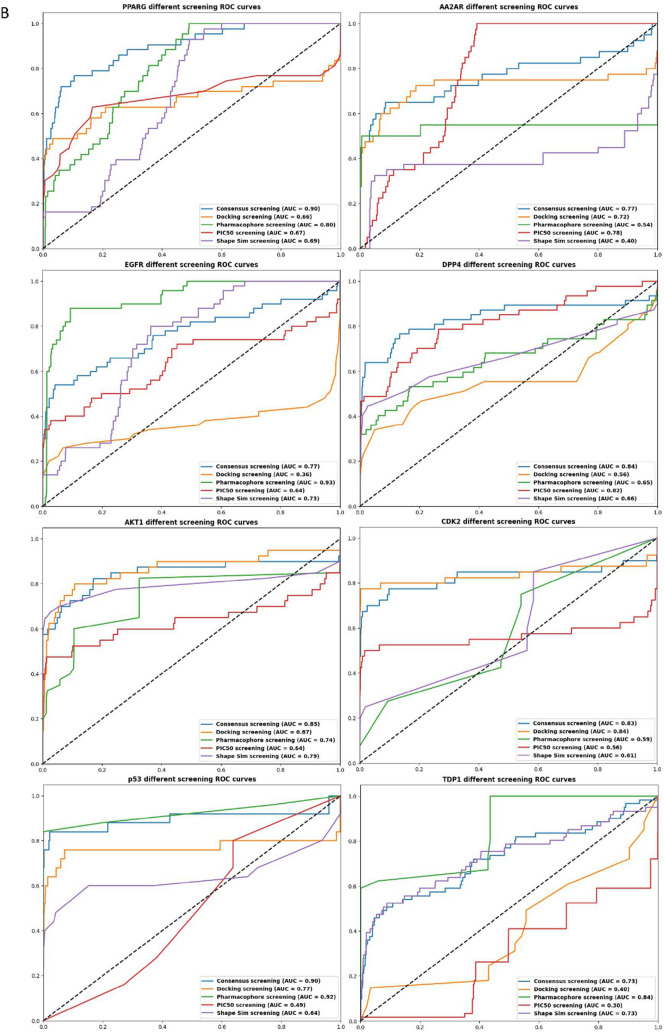
Area under the ROC curve for (**A**) consensus scoring method in protein targets involved in this study in comparison to (**B**) each target evaluated by four different screening methods; QSAR (PIC_50_), Docking, pharmacophore, and shape similarity screenings in comparison with the consensus scoring

In evaluating various screening methods against consensus screening for different protein targets, we detailed their performance metrics, including AUC ROC, EF1%, EF5%, decoy percentage at 1%, and Boltzmann-Enhanced Discrimination of ROC (BEDROC) values, as defined in the Supplementary information and Fig. 7. For the AKT1 protein target, docking screening exhibited superior performance with an AUC ROC score of 0.87, marginally higher than the consensus score of 0.85. Similarity screening followed with a score of 0.79, while Pharmacophore and QSAR methods registered scores of 0.74 and 0.64, respectively. In terms of EF1%, Similarity screening outperformed with a score of 63.0, surpassing the consensus score of 57.5. Docking and QSAR methods both achieved 40.0, and Pharmacophore screening was lower at 22.68. BEDROC scores showed Similarity screening leading with 0.5443, above the consensus of 0.523, followed by QSAR (0.3935), Docking (0.3174), and Pharmacophore (0.224).

For the CDK2 protein, docking screening again excelled with an AUC score of 0.84, slightly above the consensus of 0.83. Similarity and Pharmacophore screenings scored 0.61 and 0.59, respectively, with QSAR trailing at 0.56. EF1% values showed Docking leading significantly with 78.12, well above the consensus of 65.0. QSAR recorded 45.36, with Similarity and Pharmacophore screenings at 25.2 and 27.72, respectively. BEDROC values for Docking and QSAR were close to the consensus score of 0.4192, at 0.4864 and 0.3203, respectively, while Similarity and Pharmacophore screenings had lower values of 0.2168 and 0.2354. This comprehensive evaluation, detailed in Supplementary Table 2, underscores the variable efficacy of screening methods across protein targets, informing their strategic application in virtual screening.

In the evaluation of DPP4 using consensus scoring, the QSAR screening method's AUC score of 0.82 is closely matched to the consensus of 0.84, with Pharmacophore and Similarity methods yielding lower scores of 0.65 and 0.66, respectively, and docking the lowest at 0.56. For EF1%, QSAR and consensus both achieve 46.81, with Similarity at 36.17, Pharmacophore at 31.91 and docking significantly lower at 8.51. In BEDROC scores, QSAR exceeds consensus with 0.4893 versus 0.4559, followed by Pharmacophore and Similarity methods at 0.381 and 0.3646, respectively, and docking considerably behind at 0.0969.

For the EGFR protein, Pharmacophore screening excels with an AUC of 0.93, exceeding the consensus of 0.77. Similarity screening is close to consensus at 0.73, with QSAR at 0.64, and docking significantly behind at 0.36. QSAR's EF1% of 30.3 is near the consensus of 34.67, with Similarity and Docking trailing at 13.86 and 14.18, respectively, and Pharmacophore notably lower at 3.96. BEDROC metrics show all methods aligning closely around the consensus of 0.6139, except for QSAR which lags at 0.3649. Refer to Fig. 7 for ROC curves of the various scoring and screening methodologies.

For the AA2AR, the QSAR screening method achieved an AUC of 0.78, marginally higher than the consensus of 0.77, followed by Docking at 0.72. Pharmacophore screening recorded a lower AUC of 0.54, with Similarity trailing at 0.4. In the EF1% evaluation, Pharmacophore led with 50.4, above the consensus of 45.36, and docking at 42.84, while QSAR and Similarity both reported 0.0. BEDROC scores for Pharmacophore and Docking were close to the consensus of 0.4401, at 0.3962 and 0.3974, respectively.

In contrast, for the p53 protein, Pharmacophore screening achieved the highest AUC of 0.93, slightly above the consensus of 0.90, with Docking at 0.77 and Similarity at 0.64. QSAR was notably lower at 0.49. Pharmacophore screening exhibited outstanding EF1% performance at 88.96, surpassing the consensus of 76.82. In BEDROC metrics, Pharmacophore again led with 0.4661, exceeding the consensus of 0.4336, followed by Docking and Similarity at 0.3553 and 0.2952, respectively, and QSAR at 0.1445.

In the case of the PPARG protein, the Pharmacophore screening method achieved an AUC ROC of 0.80, near the consensus of 0.90, with Similarity, QSAR, and Docking methods following at 0.69, 0.67, and 0.66, respectively. In EF1%, Docking led with 48.67, exceeding the consensus of 42.35. Docking also topped the BEDROC metric with 0.3135, surpassing the consensus of 0.2896, with Similarity and QSAR at 0.1354 and 0.2372, respectively. Regarding the TDP1 protein, Pharmacophore screening outperformed with an AUC of 0.84, above the consensus of 0.73. Similarity matched the consensus at 0.73, while Docking and QSAR lagged with 0.4 and 0.3, respectively. For BEDROC, Pharmacophore significantly led with 0.2319, doubling the consensus of 0.1184, with Similarity and Docking at 0.1271 and 0.0623, and QSAR at 0.0163, indicating a marked disparity in the early detection of actives across screening methods.

### The consensus holistic scoring in comparison to other consensus virtual screening methods

A comparative analysis of three consensus docking approaches reveals distinct advantages and disadvantages. Houston and Walkinshaw [6] demonstrated improved pose prediction accuracy (82% success rate) and reduced false positives by integrating multiple docking programs, albeit with increased computational costs and potential rise in false negatives. Besides, Ochoa, Palacio-Rodriguez [10] introduced a score-based consensus docking approach with higher success rates in pose prediction and consideration of biological target flexibility, but its efficacy may depend on individual docking program performance and could introduce biases toward certain molecules or poses. The pose rank consensus (PRC) method [11], significantly improves systematic performance and hit rates at minimal computational cost, yet its effectiveness relies on individual docking program performance and may have limitations in scenarios with few ligands or underperforming target proteins. Studies indicate that increasing time allocated for consensus docking calculations may not significantly improve method performance, highlighting nuanced trade-offs between accuracy, computational efficiency, and inherent limitations of consensus docking in virtual screening [58].

The combined use of ligand- and structure-based methodologies in computer-aided drug design optimizes chemical and biological data integration, enhancing efficacy through synergistic exploitation of their respective advantages while mitigating individual drawbacks. This integrated approach typically outperforms standalone methods, especially when employing parallel or other integrated techniques to automate and streamline virtual screening processes [15]. However, challenges persist, including the subjective and intricate nature of sequential approach selection, the complexity of method combination in parallel strategies, and limitations in accurately predicting future virtual screening performance through retrospective analyses. Prospective assessments, though more indicative of method efficacy in identifying diverse new hits, demand significantly greater resources and expertise for execution [59].

Swann, Brown [17] devised a novel consensus method merging structure-based and ligand-based screening into a unified probabilistic framework, demonstrating superior performance compared to individual metrics. This approach integrates comprehensive chemical and structural data, enhancing the diversity of identified active compounds and offering a fresh perspective on chemical similarity for drug discovery. Despite its transformative potential in virtual screening, challenges arise from the complexity of developing and validating Probability Assignment Curves (PACs), potentially restricting accessibility to researchers without computational expertise. Furthermore, the method’s efficacy depends on data quality, necessitating caution regarding generalizability and advocating for inclusive tools or guidelines to improve accessibility. Extensive validation efforts underscore concerns regarding dataset biases, highlighting the need for broader validation to ensure method robustness and mitigate overfitting risks.

The consensus holistic scoring method showcased in this study outperforms singular methodologies in identifying potential hit compounds across diverse protein targets. Introduction of the “w_new” metric enhances drug discovery efficacy by refining ML model rankings, albeit without consistently yielding optimal ROC curves. Nevertheless, it effectively prioritizes compounds with higher experimental activity, ensuring a robust screening process. Validation against biases between active compounds and decoys enhances prediction reliability. However, the method primarily serves as a scoring tool for refining true positives and does not offer insights into binding pose predictions. Integration of multiple screening methods and ML models demands substantial computational resources and expertise, along with labor and time-intensive validation and tuning for each target-specific ML model.

## Discussion

Combining diverse methodologies in drug discovery yields comprehensive insights into ligand-receptor interactions, crucial for designing potent binders. Molecular docking predicts binding affinity and ligand orientation in proteins, unveiling interaction insights. Pharmacophore modeling identifies critical features for spacial arrangement required for binding, guiding enhanced compound design. 3D-QSAR analysis quantitatively links ligand structure to biological activity, enabling activity predictions for new compounds [60]. Furthermore, the value of molecular similarity in drug discovery becomes apparent when integrating 2D and 3D shape similarity methods, which contribute significantly to a more comprehensive workflow for identifying molecules with similar structures and properties [61]. Integrating these methods offers a holistic view, elucidating key structural elements and their impact on activity. This integrated approach ensures precise predictions, empowering rational design and optimization of novel drug candidates.

Based on our analysis, the incorporation of weighted machine learning algorithms streamlined the identification of the optimal model among the twelve machine learning models introduced in this study, which encompass commonly-utilized ML models. This coding framework holds applicability across a wide spectrum of applications and can readily integrate the novel “w_new” formula into various contexts, particularly within continuous regression models, whether applied to virtual screening or other domains. The amalgamation of three key performance enhancers, namely error reduction, R^2^ enhancement across training and validation sets, and mitigation of overfitting risks by minimizing the disparity between R^2^ values in training and validation, represents, to the best of our knowledge, a novel conceptual advance.

In this investigation, we devised a streamlined approach for the examination of active and decoy distribution in the datasets, intending to identify bias and accurately evaluate the performance metrics of models. A three-stage workflow was developed for dataset validation, including quantification of physicochemical properties, diversity analysis through fragment fingerprints, and the graphical depiction of compound distributions using 2D PCA. This methodology not only addressed biases from uneven physicochemical property distributions and analogue bias but also illustrated structural diversity. The results, supported by comparisons with Maximum Unbiased Validation (MUV) datasets, indicated a high degree of similarity in distribution patterns, except for specific datasets with unique compositions. The diversity analysis further underscored the methodological strength, showing a balanced chemical class diversity and an insightful disparity in diversity ranks towards actives. This comprehensive approach, marked by a meticulous assessment of physicochemical properties and innovative use of similarity mapping and PCA, contributed to a more precise evaluation of the chemical space and dataset biases.

The study explores the factors impacting w_new and how model complexity interacts with performance metrics. Correlation analyses reveal positive correlations between w_new and R^2^-training and R^2^-validation, while error metrics like MAE, RMSE, and MSE negatively correlate with w_new. Multiple linear regression reveals that among the considered variables, R^2^-validation and RMSE most significantly affect w_new. Overall, hyperparameters can either increase or decrease model complexity depending on the specific model and parameter. Besides, the models in this study consistently favor simplicity, which enhances interpretability, computational efficiency, and robustness in data-scarce scenarios, making them suitable for diverse applications.

Across all models, the average external validation R^2^ value is ~ 0.724, indicating a moderate to high performance with a standard deviation of 0.088, highlighting significant variability across models. The R^2^ values range from 0.586 to 0.905. The GPCR protein AA2AR, using the pharmacophore scoring method with the 'KNN' machine learning model, achieved the highest external validation R^2^ of 0.905, demonstrating excellent predictivity with R^2^-train of 0.999 and R^2^-val of 0.88. In contrast, the protein p53, utilizing the 2D fingerprint shape similarity method with the ‘Adaboost’ model, showed the lowest R^2^-ext of 0.586, despite a significantly high R^2^-train of 0.969 and R^2^-val of 0.691, suggesting limitations in generalizability possibly due to dataset specifics, overfitting, or inherent protein characteristics.

In the context of the enrichment studies, it is of note that the area under the ROC curve achieved via consensus screening within the framework of the AA2AR receptor exhibits a performance level closely comparable to that of the QSAR screening, as expounded upon in the previous section. This modest augmentation in the ROC curve’s AUC assumes negligible significance when we ascertain that the initial active compound in the dataset, CHEMBL1093479, attains prioritization after an extensive cohort comprising 91 decoy compounds within QSAR screening. Meanwhile, in the consensus scoring, the first seven positions are occupied by active compounds, manifesting potency levels extending up to a PIC_50_ value of 10. This observation receives additional corroboration through the inclusion of enrichment metrics delineated within Supplementary Table 2. These metrics encompass the BEDROC, along with the percentages denoting the early fractions (EF1% and EF5%), as well as the fraction of decoys at the 1% threshold.

A parallel scenario unfolds in our evaluation of the EGFR protein target. In the domain of consensus scoring, the top four compounds are identified as active against EGFR, exhibiting PIC_50_ values ranging from 6.77 to 9.25. In contrast, the pharmacophore screening for EGFR, yielding a notably higher ROC value of 0.93, positions the first active compound, CHEMBL451513, and the second active compound, CHEMBL516022, at significantly lower ranks within the entire compound pool in the enrichment study, specifically at the 43rd and 47th positions, respectively. Remarkably, among the top-ranked compounds prominently enriched in the top ten ranks in the consensus scoring results for EGFR, are compounds such as CHEMBL63786, CHEMBL176582, and CHEMBL460723, each exhibiting the highest PIC_50_ values within the dataset, measuring 11.52, 11, and 9.25, respectively.

**Fig. 8:**
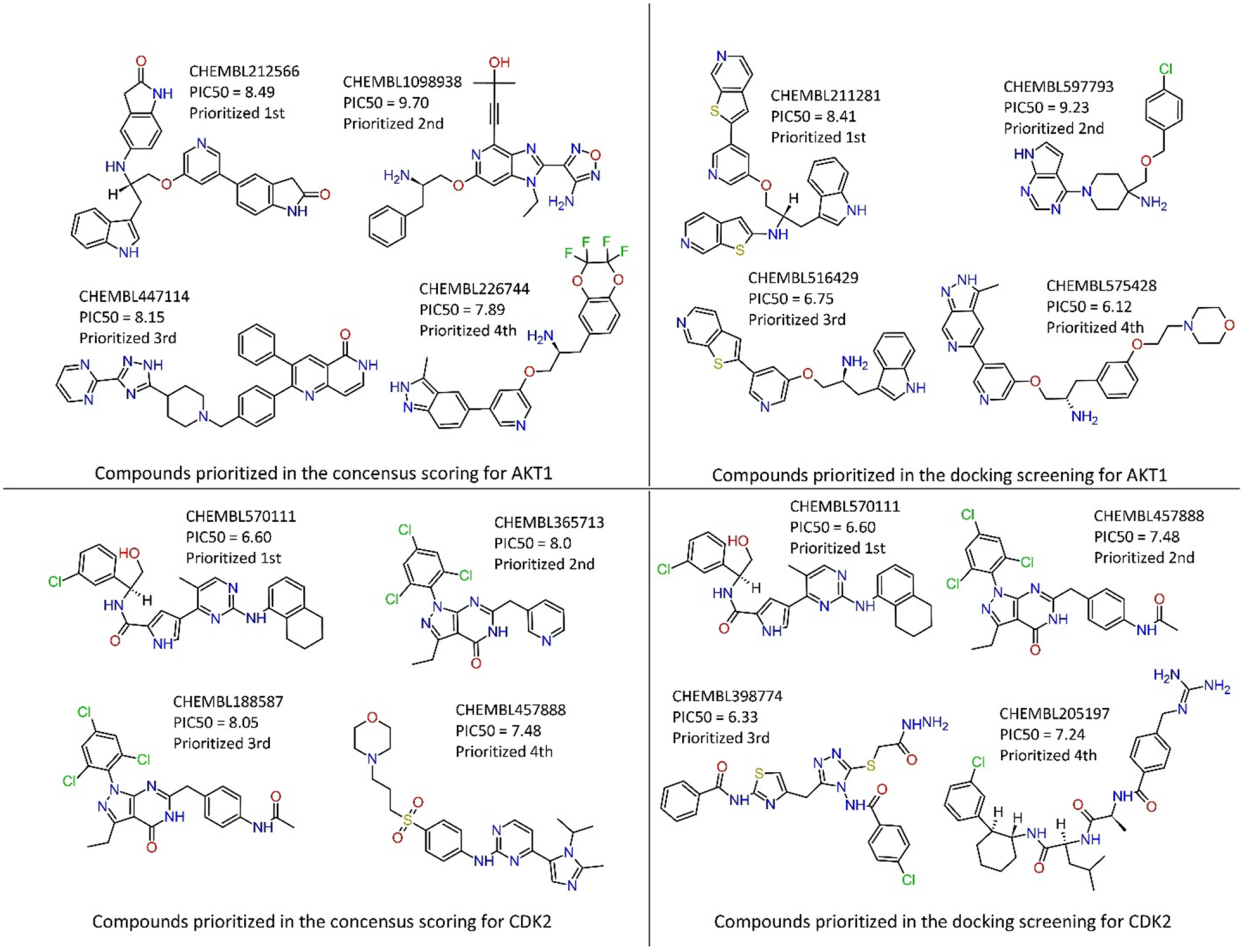
Top-ranked compounds in AKT1 and CDK2 targets in consensus and docking methodologies with their respective PIC_50_ values

Continuing within the same analytical framework, we assess the ROC AUC for the AKT1 target when comparing consensus scoring to docking screening. While the AUC values appear to exhibit minimal disparity, a more discerning examination reveals that the metrics of EF1% and BEDROC unequivocally favor the consensus scoring approach. Furthermore, when we consider additional metrics such as EF5% and the decoy percentage at the initial 1%, it becomes evident that shape similarity screening outperforms the docking method in this context. It is crucial to emphasize that a singular performance metric cannot definitively establish the superiority of one scoring or screening method over another. Hence, a comprehensive evaluation must also consider the prioritization of active compounds within each method. In Fig. 8, we observe that compounds identified as top-ranked by the consensus scoring method exhibit superior PIC_50_ values compared to those identified by the docking approach. Specifically, CHEMBL212566 and CHEMBL1098938, top-ranked by consensus scoring, display PIC_50_ values of 8.49 and 9.70, respectively. In the same vein, the consensus scoring prominently enriches CHEMBL523586 at the 24th rank. However, within the docking approach, despite its noteworthy PIC_50_ value of 10.52, CHEMBL523586 assumes a considerably lower rank, standing at 1816th. Similarly, in the shape similarity screening, its ranking descends even further, settling at the 5037th position, thereby unveiling a substantial divergence across these methodologies. These findings underscore the multifaceted nature of our evaluation, where a holistic assessment considers not only quantitative metrics but also the prioritization of active compounds as a pivotal aspect of the screening process.

In the CDK2 screening analysis, it is notable that the enrichment metrics derived from consensus and docking screening exhibited a close alignment concerning various parameters, including AUC, EF1%, EF5%, BEDROC, and Decoy Percentage at 1%, albeit with a slight advantage observed in favor of the docking method. However, a more nuanced assessment reveals that the consensus scoring approach excelled in the prioritization of compounds with higher PIC_50_ values. This distinction is particularly evident when scrutinizing Fig. 8, which highlights the top four active compounds with PIC_50_ values ranging from 6.60 to 8.05 for consensus scoring, as opposed to a narrower range of 6.33 to 7.48 for the docking screening. Furthermore, it is noteworthy that compounds possessing the highest PIC_50_ values within the CDK2 dataset received more favorable rankings within the consensus scoring methodology compared to the docking screening. For instance, the compound with the highest PIC_50_ value, namely CHEMBL360520, attaining 9.52, was positioned at the 18th rank in the consensus scoring, while the docking method placed it considerably lower at the 3415th position. Similarly, the second top-ranked compound in terms of PIC_50_ (CHEMBL261720) within the dataset achieved a ranking of 28th in consensus scoring, while the docking method assigned it a lower ranking of 44th.

In an alternative context, the performance of consensus scoring for TDP1 demonstrated diminished robustness when compared to its efficacy in assessing other macromolecules. Notably, pharmacophore screening exhibited markedly superior performance across all evaluation metrics in contrast to the consensus screening approach. This distinctive behavior observed for the TDP1 target can be ascribed to the limited activity range present within the datasets. Intriguingly, the consensus scoring for TDP1, conducted using commercially available software as described by Moshawih, Goh [22], yielded a remarkably high AUC ROC value of ~ 0.98. This exceptional outcome can be attributed to meticulous process optimization, including the selection of an optimal model and a well-suited set of features. Additionally, in this study, the decoy pool consisted of 2700 compounds for the same dataset, introducing an added layer of complexity to the analysis. In a different context, it is noteworthy that the p53 dataset is relatively small, consisting of only 20 active compounds (and 5 external validation datasets) primarily comprising anthraquinones and chalcones. Nevertheless, the consensus methodology demonstrated exemplary performance across all enrichment metrics, mirroring the trends observed with the pharmacophore approach. Moreover, the consensus scoring for p53 was also performed using commercial software in a separate study (data not published), and the resulting AUC ROC and other pertinent metrics closely paralleled the findings reported herein, with a value of 0.90. This observation suggests that consensus scoring has the capacity to effectively identify optimal characteristics from diverse screening methodologies across a wide range of scenarios and combine them to obtain the best enrichment in virtual screening.

## Conclusion

In this investigation, we undertook a comprehensive analysis involving eight diverse protein targets across various functional categories. Our primary objective was to evaluate the efficacy of a consensus holistic virtual screening approach across heterogeneous datasets. Significantly, while the PIC_50_ values for some protein targets displayed a constrained distribution, emphasizing the limited range of activities, the shape similarity scores followed by other screenings exhibited consistent and widespread patterns across all targets. Particularly, when combined with all of the screening methods through a consensus approach, it is expected to emerge as a potent strategy, demonstrating that consensus scoring selects the most favorable aspects from multiple screening metrics.

This investigation integrated a novel methodology for analyzing active and decoy distribution biases in datasets, which significantly impacted model performance and highlighted the importance of dataset validation in virtual screening. Our quest for a robust consensus scoring methodology for a holistic virtual screening led us to employ a variety of machine learning models devised with a novel formula that amalgamates five performance metrics into a unified measure called w_new. The greater weight assigned (w_new) signifies a robust model performance, characterized by higher R^2^-training and -validation scores, reduced MAE, RMSE, and MSE values, and minimized disparity between R^2^-train and -validation and vice versa. This comprehensive study unveiled a spectrum of performance metrics among different models employed.

In our endeavor to elucidate the factors influencing w_new values and assess the impact of model complexity on performance metrics, we conducted an exhaustive analysis. Our investigation revealed that R^2^-validation and RMSE are pivotal factors influencing w_new, exhibiting positive and negative correlations, respectively. These findings underscore models used in this study consistently prioritize simplicity, leading to improved computational efficiency, data efficiency, and practical applicability. Furthermore, our study shed light on the nuanced relationships between w_new and various model-specific parameters, providing insights into the interplay between model complexity and performance metrics.

Overall, weighted machine learning models find utility across diverse domains and are not restricted to virtual screening, where the primary objective is the identification of optimal, high-performing, and resilient models. Besides, this comprehensive analysis underscores the importance of considering not only quantitative metrics but also the prioritization of active compounds, which can vary significantly across different methods when choosing screening and scoring methodologies. This analysis emphasizes the effectiveness of consensus scoring as a crucial virtual screening technique, often yielding superior performance in terms of AUC, early detection of actives, prioritizing compounds with the highest biological activities, or a combination of these factors. These findings contribute significantly to advancing our understanding of screening techniques' performance in diverse protein target contexts, ultimately enhancing the effectiveness of virtual screening approaches.

### Supplementary Information

Supplementary Material 1.

Supplementary Material 2.

Supplementary Material 3.

## Data Availability

The code snippets developed and other code implementations utilized in this study can be accessed via the following GitHub link: https://github.com/Saeedmomo/Consensus_Holistic_Virtual_Screening.git. Furthermore, the datasets used in this research are also accessible within the same GitHub repository.
