# Supplementary material for: Consensus holistic virtual screening for drug discovery: a novel machine learning model approach
Source: J Cheminform. 2024 May 28;16:62. doi: 10.1186/s13321-024-00855-8 (PMC11134635; doi:10.1186/s13321-024-00855-8)
Supplement: Supplementary file 1 — Supplementary Material 1. [file 13321_2024_855_MOESM1_ESM.docx]

**Supplementary Information:**

**Consensus Holistic Virtual Screening for Drug Discovery: A Novel Machine Learning Model Approach**

Supplementary Table 1. Diverse Machine Learning Models: An Overview of Model Parameters, Feature Counts, PCA Components, and Cross-Validation Strategies for Scoring Methods Across Various Target Proteins.

| Target  protein  Scoring method | | ML model | No. PCA/Features | Cross-validation | Model parameters | | | |
| --- | --- | --- | --- | --- | --- | --- | --- | --- |
| AA2AR | PIC_50_ | SVR RBF | 190 F. | 10 | C= 2.7 | γ= 0.3 | - | - |
|  | pharm | KNN | 90 F. | 10 | K=5 | Weight= distance | - | - |
|  | Docking | Elastic net Reg. | 20 pca | 10 | α= 0.3 | L1= 0.99 | - | - |
|  | Similarity | Nu-SVR linear | 180 F. | 10 | C= 2 | Kernel: linear | Nu= 0.4 | - |
| TDP1 | PIC_50_ | Dec. Tree | 13 pca | 10 | Max Depth = 8 | Min split = None | Min leaf = 1 | - |
|  | pharm | Dec. Tree | 10 pca | 10 | Max Depth = 10 | Min split = 4 | Min leaf = 2 | - |
|  | Docking | Dec. Tree | 20 pca | 10 | Max Depth = 6 | Min split = 3 | Min leaf = 2 | - |
|  | Similarity | Elastic net Reg. | 15 pca | 10 | α= 0.0001 | L1= 0.89 | - | - |
| EGFR | PIC_50_ | Random forest | 10 pca | 10 | Max Depth = 8 | No. of estimator= 9 | Min leaf = 6 | Min split = 3 |
|  | pharm | Adaboost | 12 pca | 10 | No. of estimator= 100 | Learn rate= 1 | Loss: sq | - |
|  | Docking | Adaboost | 12 pca | 5 | No. of estimator= 50 | Learn rate= 1 | Loss: sq | - |
|  | Similarity | Adaboost | 12 pca | 10 | No. of estimator= 50 | Learn rate= 1 | Loss: Expo | - |
| Akt1 | PIC_50_ | SVR RBF | 20 pca | 10 | C= 0.9 | γ= 0.4 | - | - |
|  | pharm | KNN | 20 pca | 10 | K= 1 | Weight= distance | - | - |
|  | Docking | SVR RBF | 10 pca | 10 | C = 5.5 | γ= 0.1 | - | - |
|  | Similarity | Adaboost | 10 pca | 8 | No. of estimator= 100 | Learn rate= 1 | Loss: Sq | - |
| DPP4 | PIC_50_ | Nu-SVR  RBF | 17 pca | 12 | C= 2.9 | Kernel: RBF | Nu= 0.4 | - |
|  | pharm | Nu-SVR  RBF | 160 F. | 10 | C= 0.3 | Kernel: RBF | Nu= 0.7 | - |
|  | Docking | Adaboost | 8 pca | 8 | No. of estimator= 150 | Learn rate= 1 | Loss: Sq | - |
|  | Similarity | Adaboost | 12 pca | 10 | No. of estimator= 150 | Learn rate= 1 | Loss: Sq | - |
| CDK2 | PIC_50_ | Random Forest | 13 pca | 10 | Max Depth = 4 | No. of estimator= 7 | Min leaf = 57 | Min split = 10 |
|  | pharm | Dec. Tree | 15 pca | 10 | Max Depth = 3 | Min split = 6 | Min leaf = 1 | - |
|  | Docking | SVR RBF | 20 pca | 10 | C = 0.8 | γ= 0.1 |  |  |
|  | Similarity | Dec. Tree | 13 pca | 8 | Max Depth = 3 | Min split = 1 | Min leaf = 3 | - |
| PPARG | PIC_50_ | Adaboost | 15 pca | 10 | No. of estimator= 100 | Learn rate= 1 | Loss: Sq | - |
|  | pharm | Gradient boosting | 10 pca | 12 | No. of estimator= 100 | Max Depth = 10 | Min split= 2 | Subsample= 0.75 |
|  | Docking | Nu-SVR  RBF | 18 pca | 8 | C= 2 | Kernel: RBF | Nu= 0.7 | - |
|  | Similarity | KNN | 8 pca | 12 | K= 4 | Weight: Distance | - | - |
| P53 | PIC_50_ | Dec. Tree | 25 pca | 10 | Max Depth = 5 | Min split = 3 | Min leaf = 5 | - |
|  | pharm | Adaboost | 20 pca | 10 | No. of estimator= 40 | Learn rate= 1 | Loss: Sq | - |
|  | Docking | Elastic net Reg. | 20 pca | 10 | α= 0.014 | L1= 0.99 | - | - |
|  | Similarity | Adaboost | 10 pca | 10 | No. of estimator= 100 | Learn rate= 1 | Loss: Sq | - |


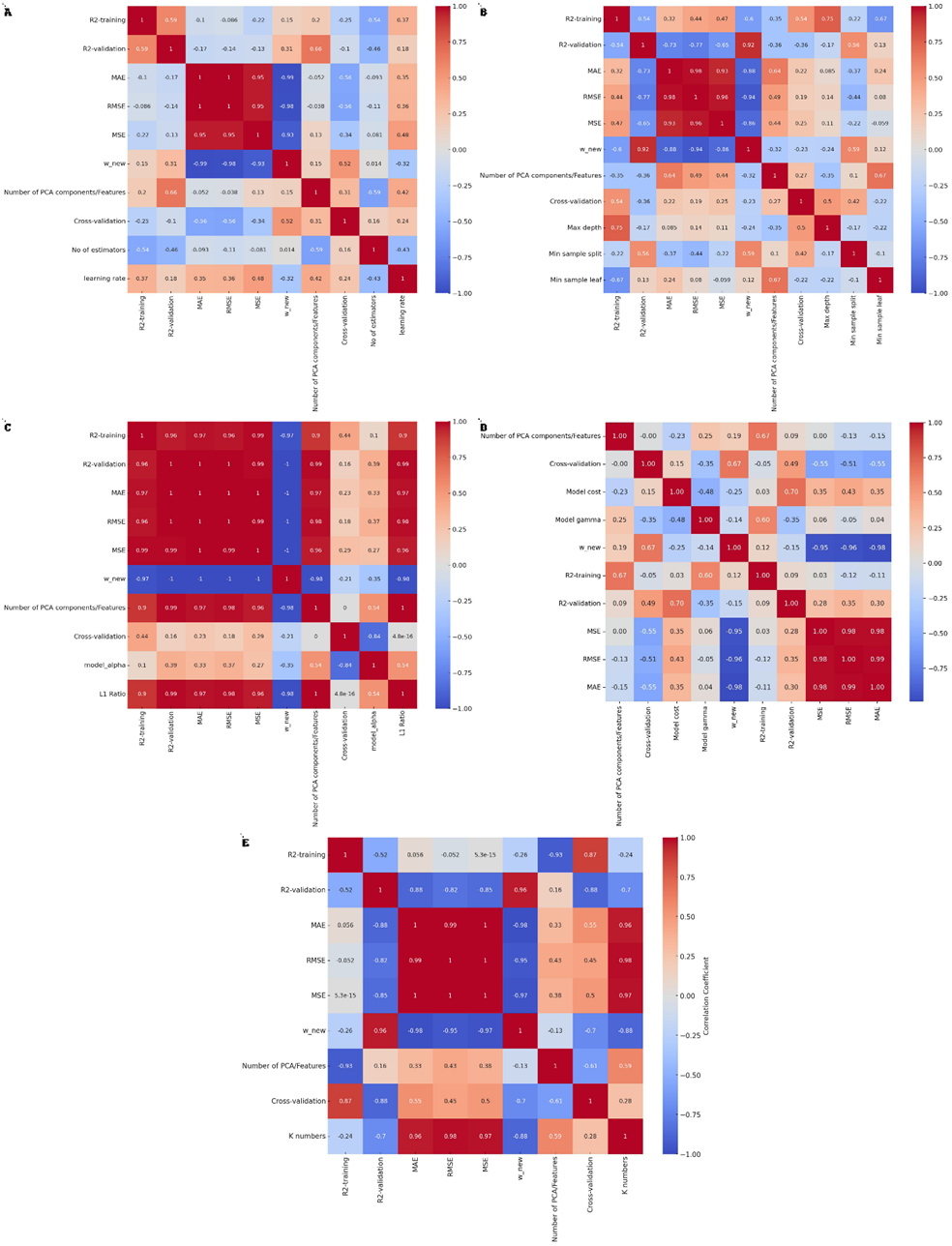


**Supplementary Figure 1.** The correlation heatmaps for the main models employed in this study with the correlation between specific model parameters, CV, and PCA/features components with performance metrics A) Adaboost, B) Decision Tree C) Elastic Net regression D) SVR models E) KNN.

​**Supplementary Table 2.** Major enrichment metrics; AUC, EF1%, EF5%, BEDROC, and Decoy Percentage at 1% for all protein targets in different PIC_50_, Pharmacophore, Docking, and Similarity screening methods in comparison to the consensus screening.

| **AKT1** | **PIC_50__screening** | **Pharmacophore_screening** | **Docking_screening** | **Similarity_screening** | **Consensus_scoring** |
| --- | --- | --- | --- | --- | --- |
| AUC | 0.64 | 0.74 | 0.87 | 0.79 | 0.85 |
| EF1%: | 40.32 | 22.68 | 40.32 | 63.0 | 57.50 |
| EF5%: | 9.5 | 7.0 | 13.5 | 13.5 | 12.50 |
| BEDROC | 0.3935 | 0.2240 | 0.3174 | 0.5443 | 0.5230 |
| Decoy Percentage at 1%: | 68% | 82% | 68% | 50% | 54.0% |
| **CDK2** | **PIC_50__screening** | **Pharmacophore_screening** | **Docking_screening** | **Similarity_screening** | **Consensus_scoring** |
| AUC | 0.56 | 0.59 | 0.84 | 0.61 | 0.83 |
| EF1%: | 45.36 | 27.72 | 78.12 | 25.20 | 65.00 |
| EF5%: | 10.0 | 5.5 | 15.5 | 10.0 | 14.00 |
| BEDROC | 0.3203 | 0.2354 | 0.4864 | 0.2168 | 0.4192 |
| Decoy Percentage at 1%: | 64% | 78% | 38% | 80% | 48.0 |
| **DPP4** | **PIC_50__screening** | **Pharmacophore_screening** | **Docking_screening** | **Similarity_screening** | **Consensus_scoring** |
| AUC | 0.82 | 0.65 | 0.56 | 0.66 | 0.84 |
| EF1%: | 46.81 | 31.91 | 8.51 | 36.17 | 46.81 |
| EF5%: | 9.79 | 7.24 | 2.98 | 10.21 | 12.77 |
| BEDROC | 0.4893 | 0.3810 | 0.0969 | 0.3646 | 0.4559 |
| Decoy Percentage at 1%: | 56% | 70% | 93% | 66% | 56.0 |
| **EGFR** | **PIC_50__screening** | **Pharmacophore_screening** | **Docking_screening** | **Similarity_screening** | **Consensus_screening** |
| AUC | 0.64 | 0.93 | 0.36 | 0.73 | 0.77 |
| EF1%: | 30.30 | 3.96 | 14.18 | 13.86 | 34.67 |
| EF5%: | 7.62 | 14.37 | 4.10 | 2.79 | 10.78 |
| BEDROC | 0.3649 | 0.6138 | 0.5748 | 0.6136 | 0.6139 |
| Decoy Percentage at 1%: | 70% | 96.08% | 85.95% | 86.27% | 66.67% |
| **AA2AR** | **PIC_50__screening** | **Pharmacophore_screening** | **Docking_screening** | **Similarity_screening** | **Consensus_screening** |
| AUC | 0.78 | 0.54 | 0.72 | 0.40 | 0.77 |
| EF1%: | 0.0 | 50.4 | 42.84 | 0.0 | 45.36 |
| EF5%: | 2.5 | 10.05 | 10.0 | 6.0 | 11.50 |
| BEDROC | 0.0029 | 0.3962 | 0.3974 | 0.0017 | 0.4401 |
| Decoy Percentage at 1%: | 100% | 60% | 66.67% | 100% | 64.71% |
| **P53** | **PIC_50__screening** | **Pharmacophore_screening** | **Docking_screening** | **Similarity_screening** | **Consensus_screening** |
| AUC | 0.49 | 0.93 | 0.77 | 0.64 | 0.90 |
| EF1%: | 9.28 | 88.96 | 52.57 | 44.48 | 76.82 |
| EF5%: | 6.82 | 17.64 | 13.63 | 11.22 | 16.83 |
| BEDROC | 0.1445 | 0.4661 | 0.3553 | 0.2952 | 0.4336 |
| Decoy Percentage at 1%: | 92.09% | 4.3% | 43.5% | 48% | 17.39% |
| **PPARG** | **PIC_50__screening** | **Pharmacophore_screening** | **Docking_screening** | **Similarity_screening** | **Consensus_screening** |
| AUC | 0.67 | 0.80 | 0.66 | 0.69 | 0.90 |
| EF1%: | 32.84 | 20.70 | 48.67 | 16.10 | 42.35 |
| EF5%: | 6.98 | 6.49 | 9.73 | 3.26 | 12.56 |
| BEDROC | 0.2372 | 0.0696 | 0.3135 | 0.1354 | 0.2896 |
| Decoy Percentage at 1%: | 72% | 82.35% | 64.71% | 86.27% | 64.71% |
| **TDP1** | **PIC_50__screening** | **Pharmacophore_screening** | **Docking_screening** | **Similarity_screening** | **Consensus_scoring** |
| AUC | 0.30 | 0.84 | 0.40 | 0.73 | 0.73 |
| EF1%: | 1.68 | 42.14 | 6.71 | 16.17 | 19.40 |
| EF5%: | 0.33 | 8.60 | 3.61 | 8.79 | 8.47 |
| BEDROC | 0.0163 | 0.2319 | 0.0623 | 0.1271 | 0.1184 |
| Decoy Percentage at 1%: | 96.42% | 6.90% | 85.2% | 64.29% | 57.14% |

​

**Formulas:**

• **Enrichment Factor at x% (EFx%)**:

EF5% = (Hits at x% / Total compounds screened at 5%) / (Total hits / Total compounds screened)

• **BEDROC (****Boltzmann-Enhanced Discrimination of ROC)**:

BEDROC = (1/B) * Σ [e^(-α * R_i)] * (1 - R_i)

Where:

- B is a normalization factor, typically the highest possible value of Σ [e^(-α * R_i)] * (1 - R_i) in the absence of enrichment.
- α is a parameter that controls the weighting of early enrichment over the entire ROC curve.
- R_i is the fraction of the ranked list (i / N) where N is the total number of compounds.

• **Decoy Percentage at 1%**:

Decoy Percentage at 1% = (Number of decoys in the top 1% / Total compounds screened at 1%) * 100%
