# Supplementary material for: Consensus holistic virtual screening for drug discovery: a novel machine learning model approach
Source: J Cheminform. 2024 May 28;16:62. doi: 10.1186/s13321-024-00855-8 (PMC11134635; doi:10.1186/s13321-024-00855-8)
Supplement: Supplementary file 3 — Supplementary Material 3. [file 13321_2024_855_MOESM3_ESM.docx]

**Figure 2, A**


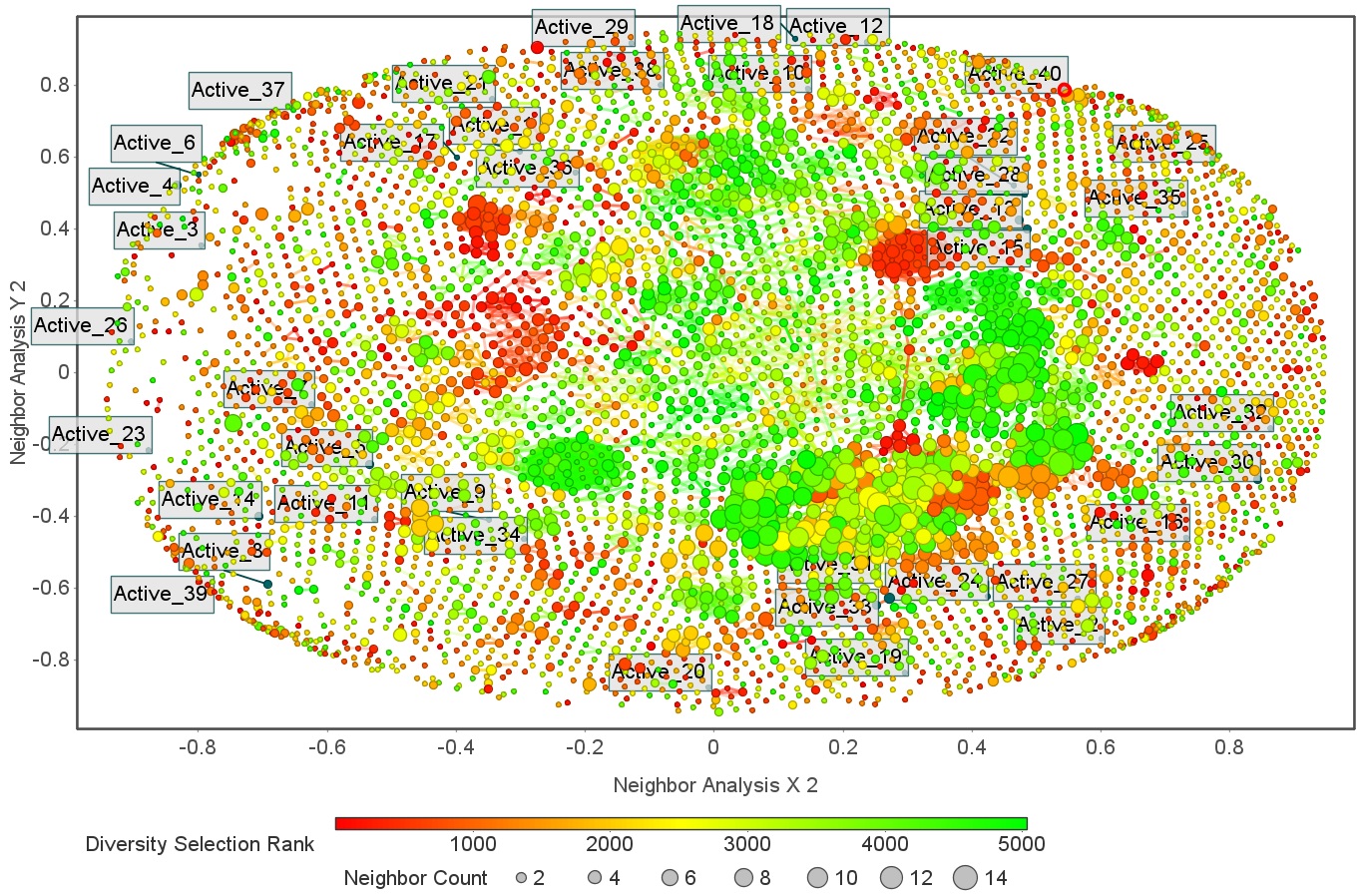


CDK2


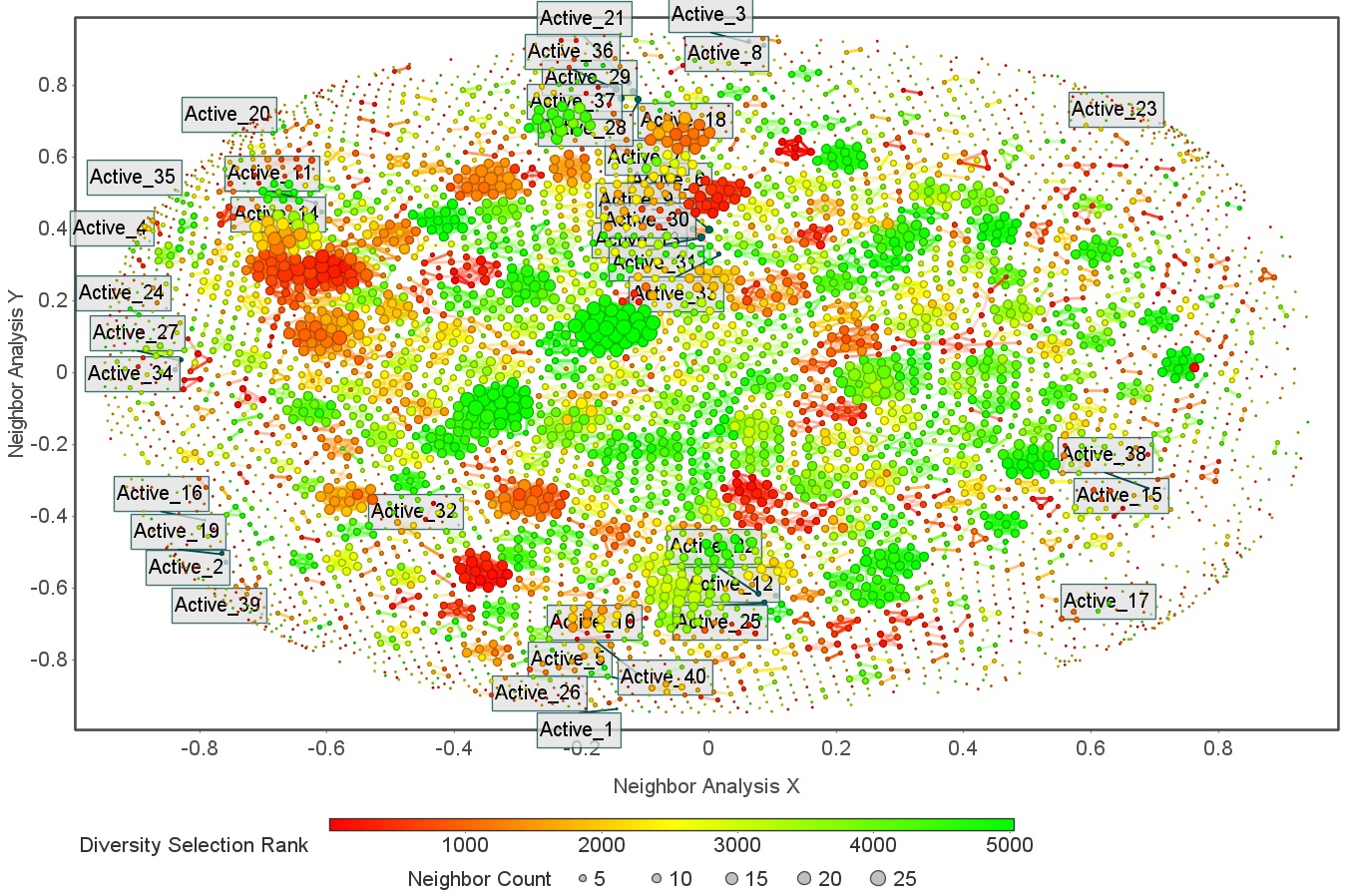


AKT1


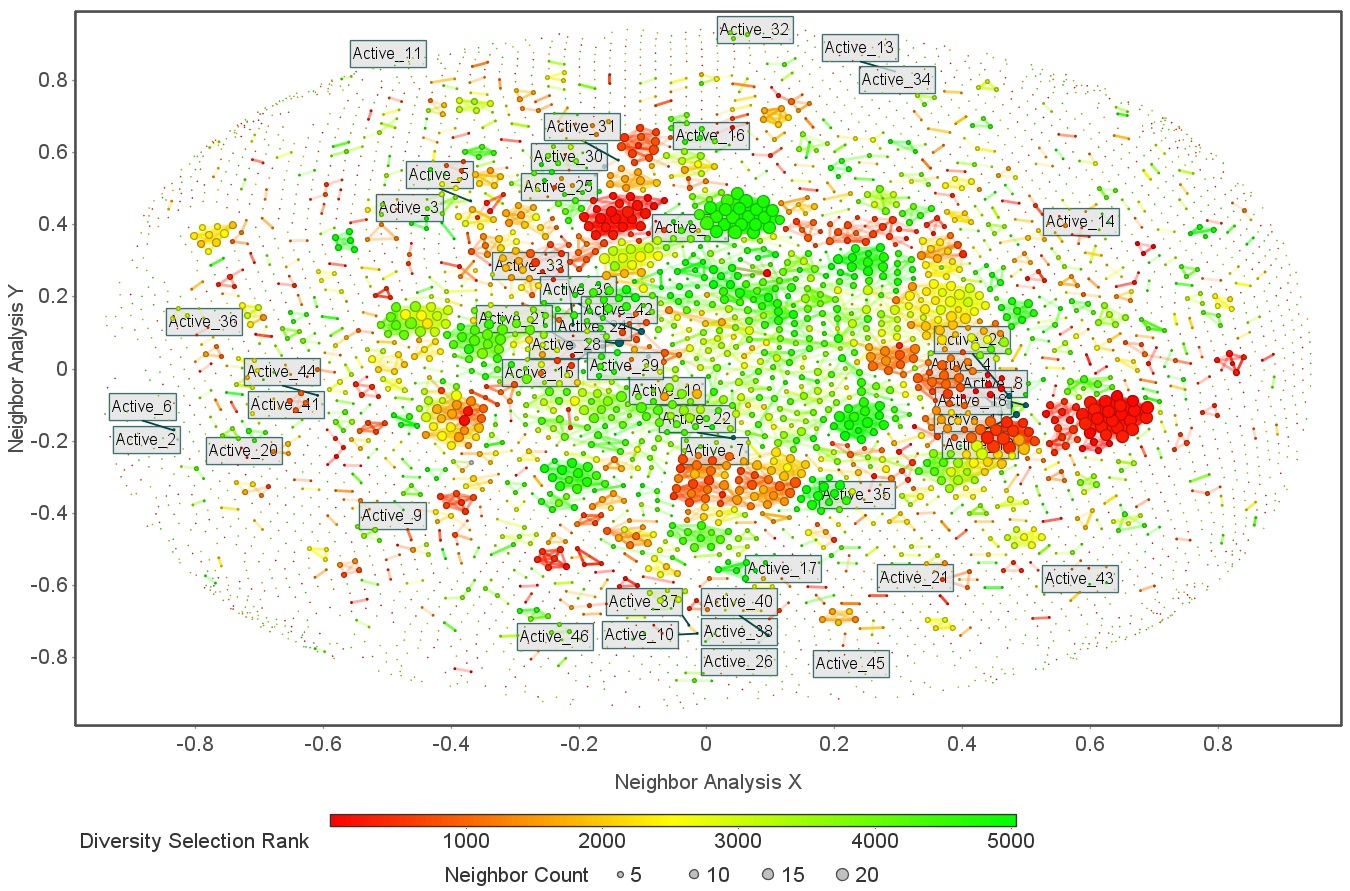


DPP4


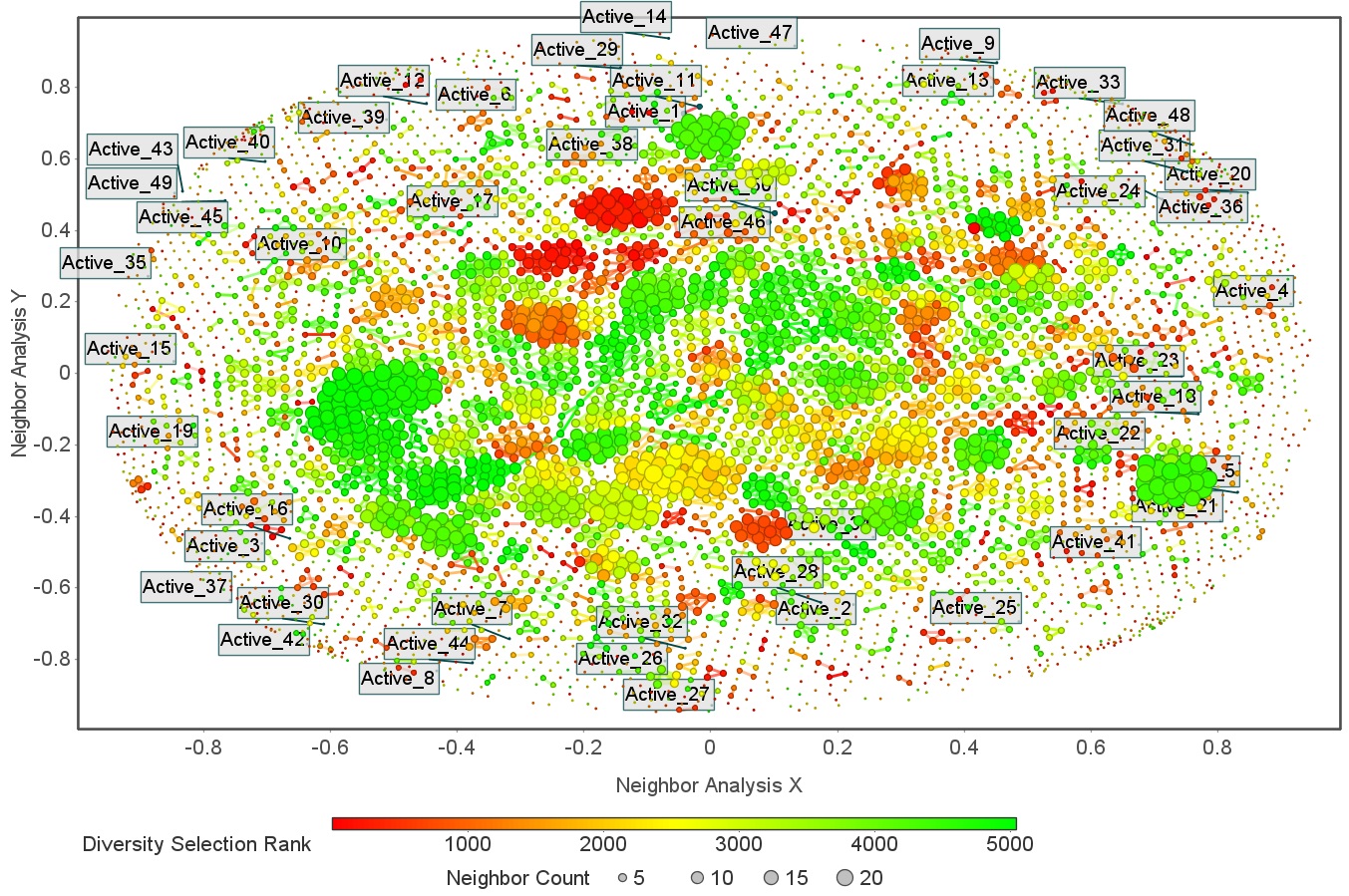


EGFR


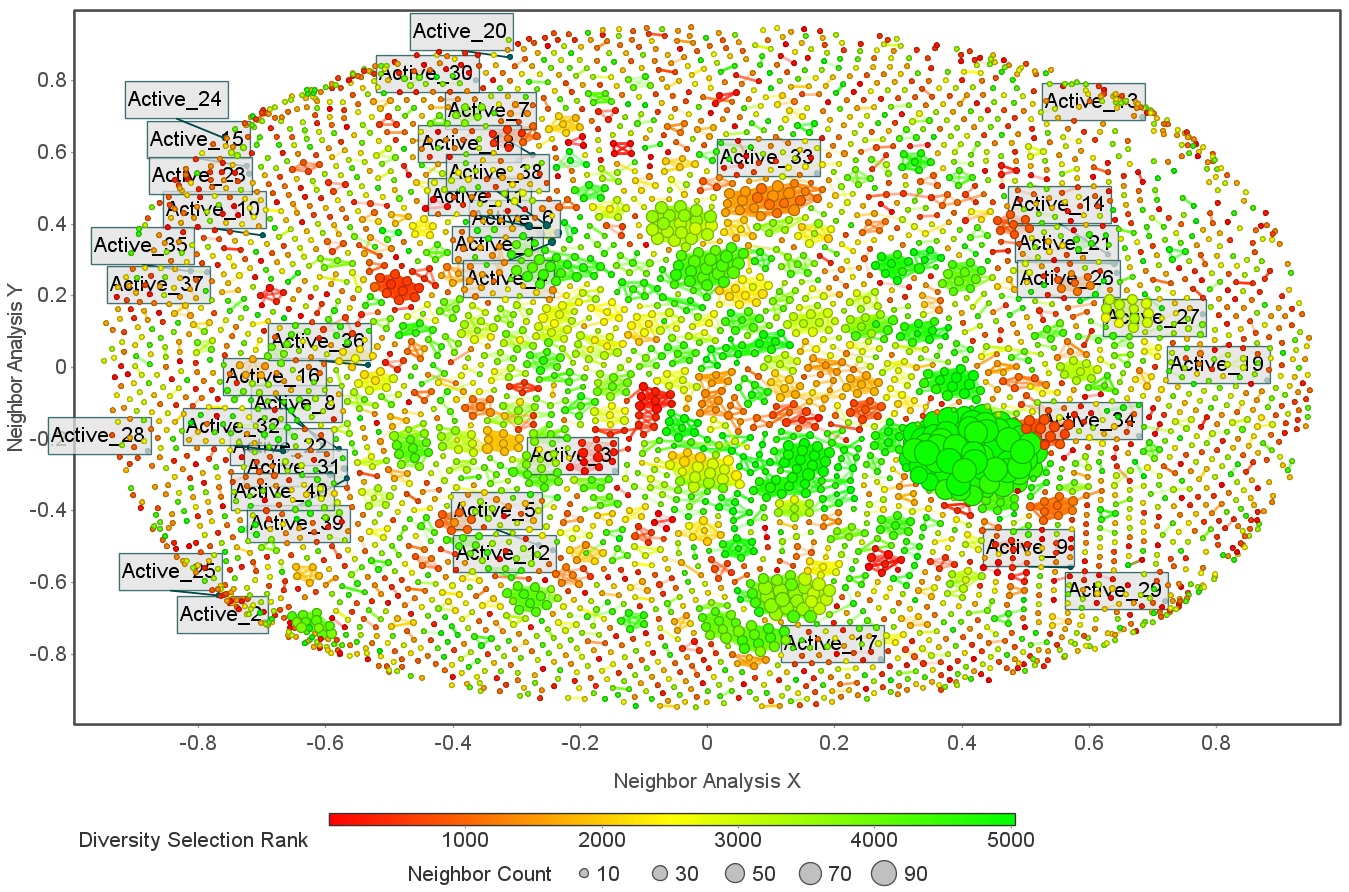


AAR2AR


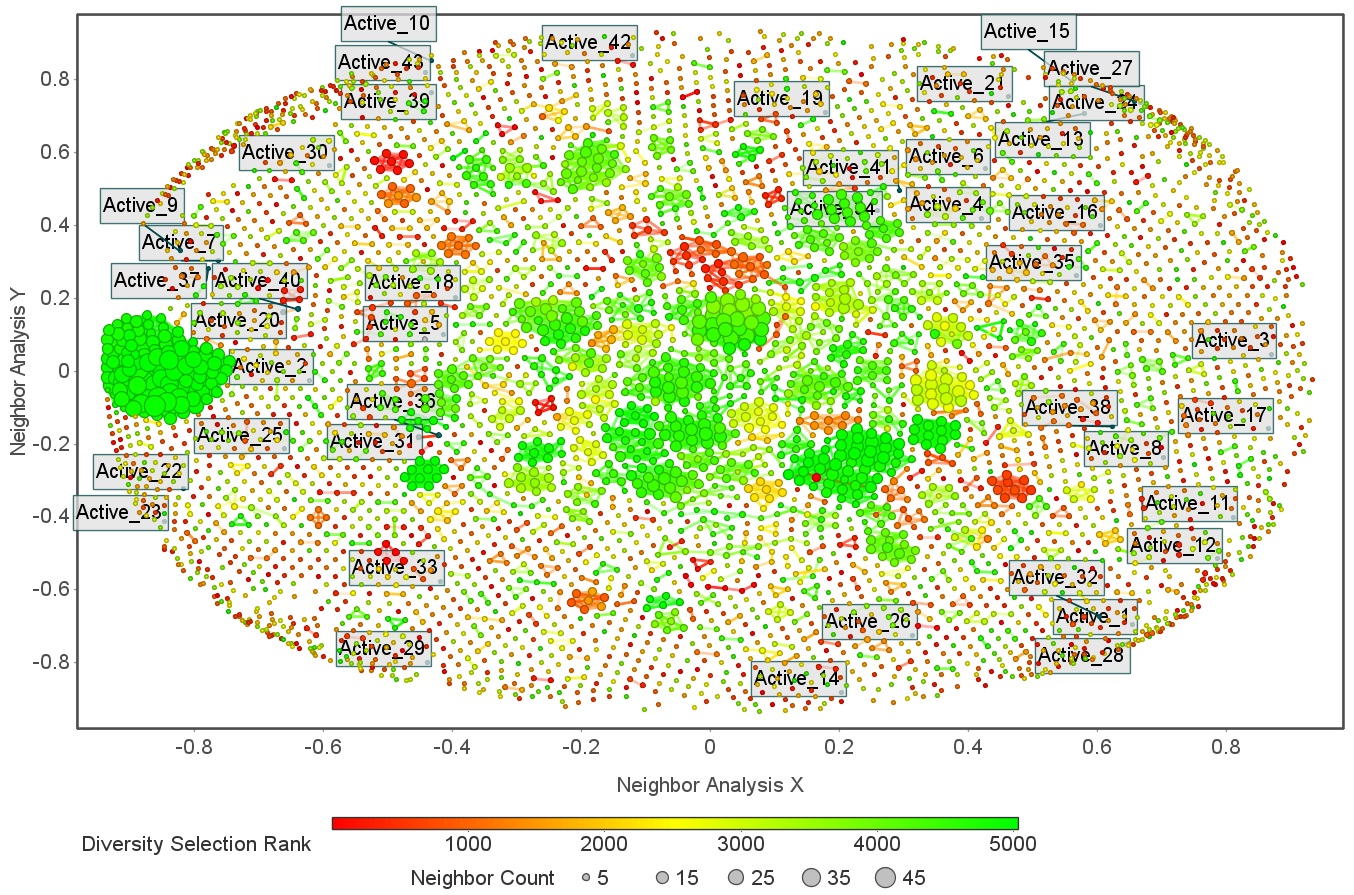


PPARG


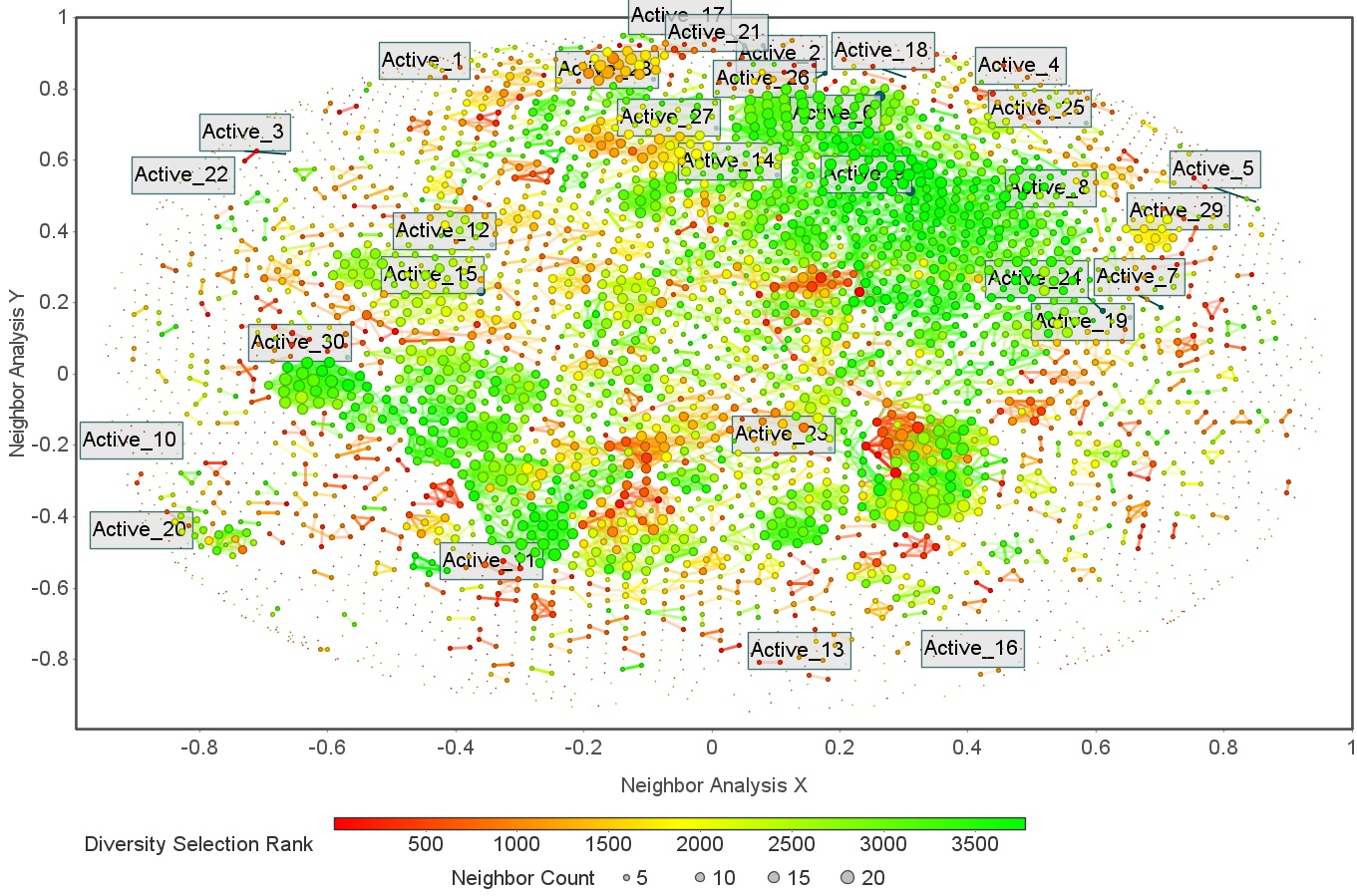


MUV-737


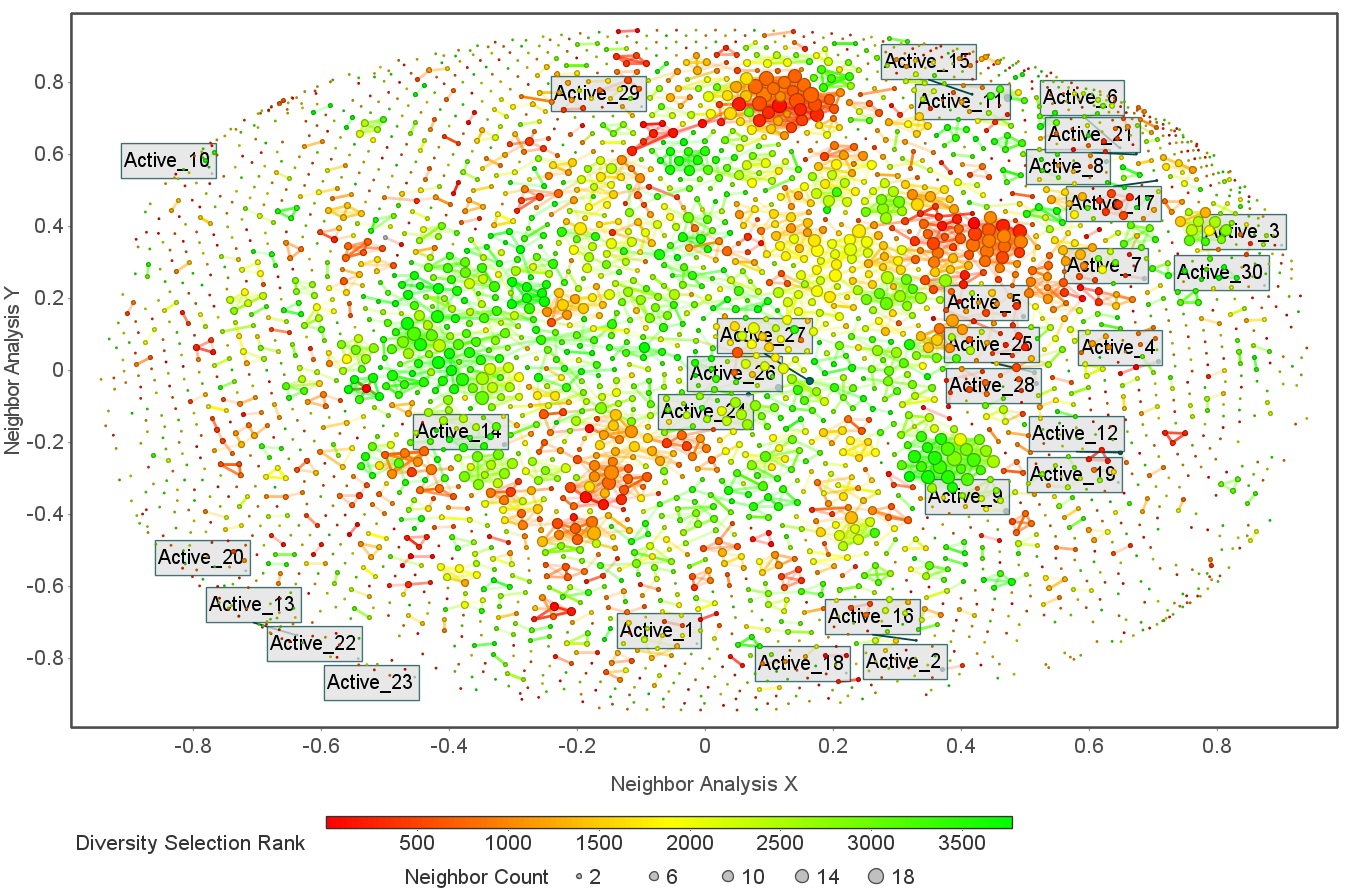


MUV-810


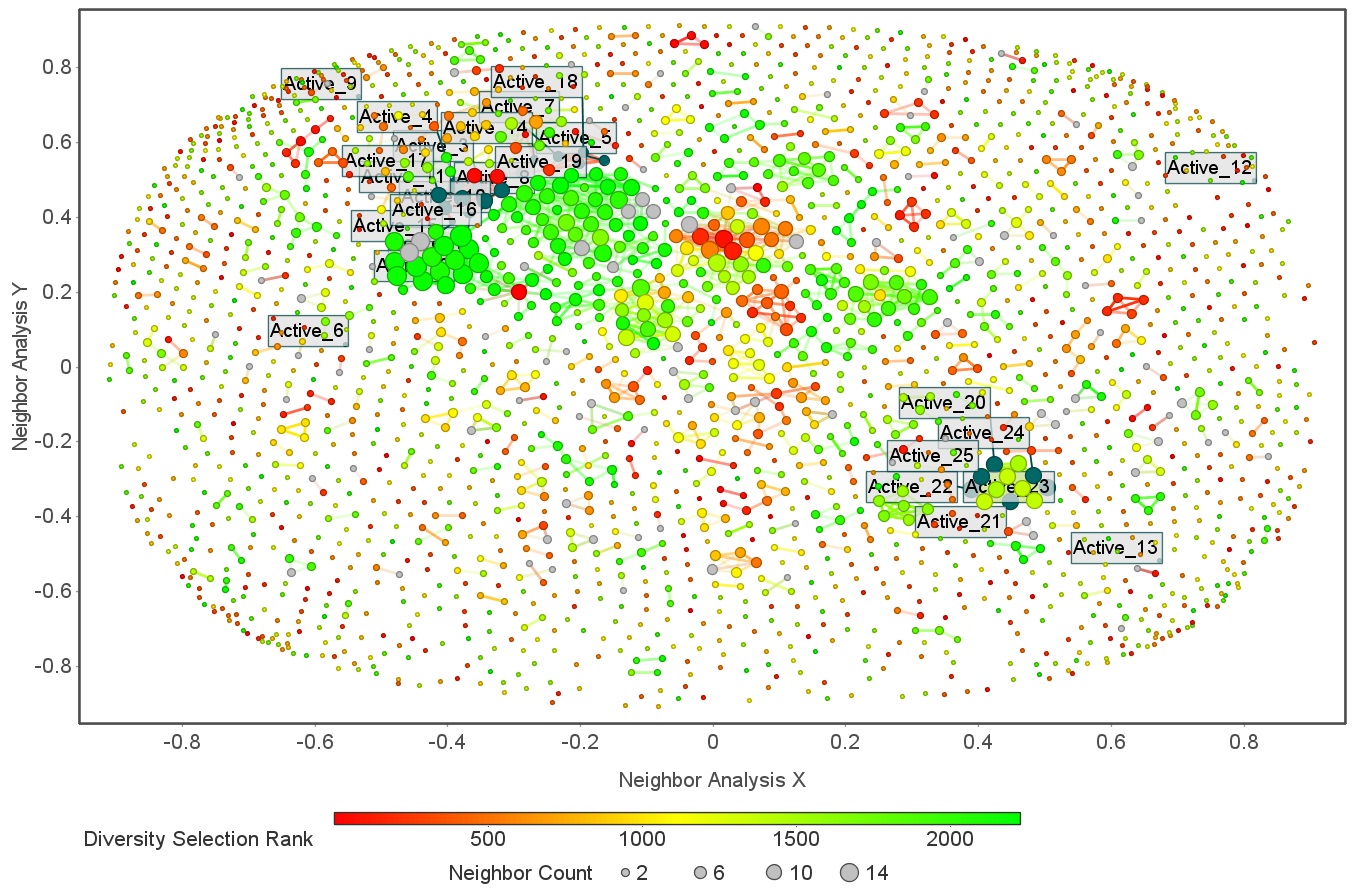


p53


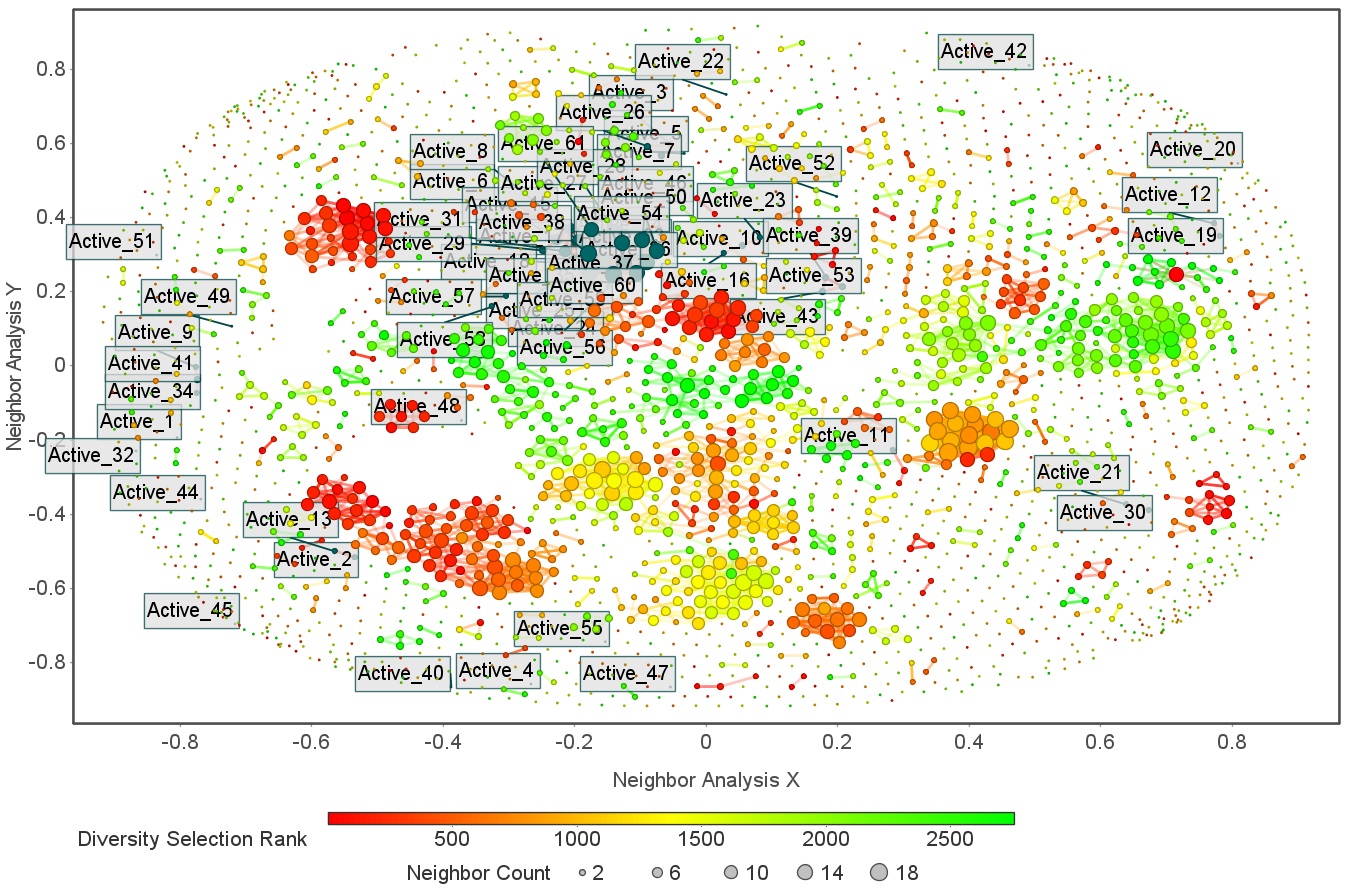


TDP1

**Figure 2, B**


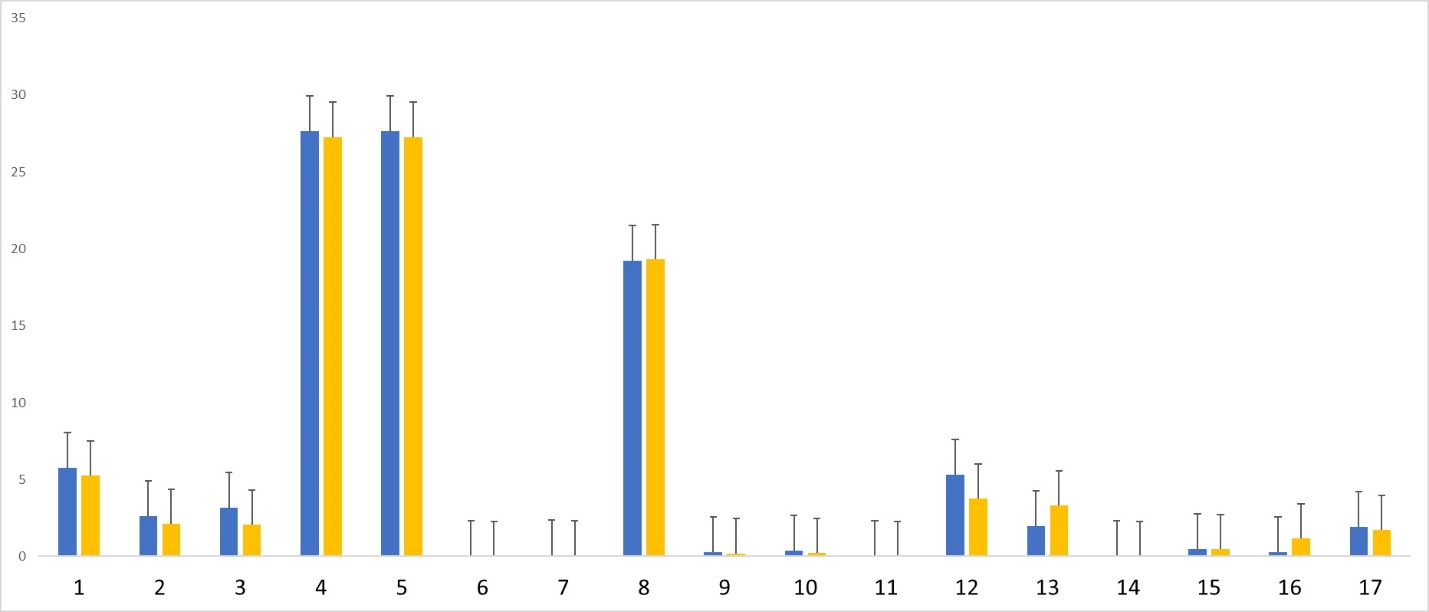


CDK2


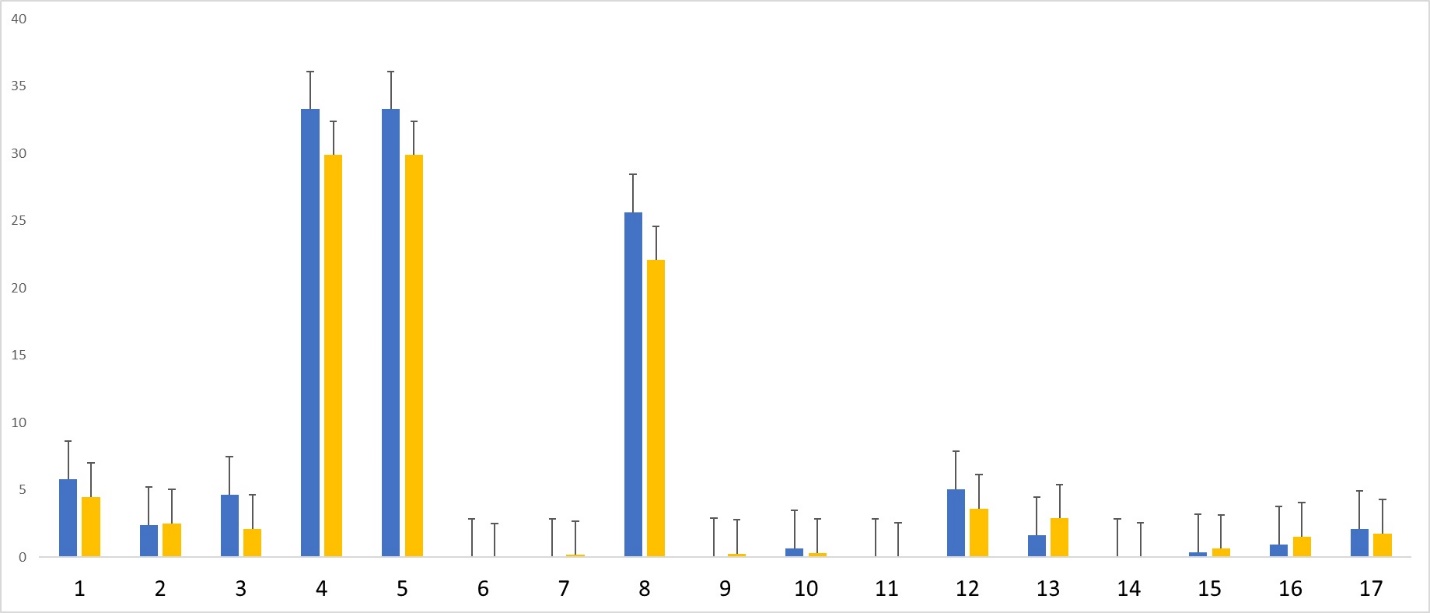


AKT1


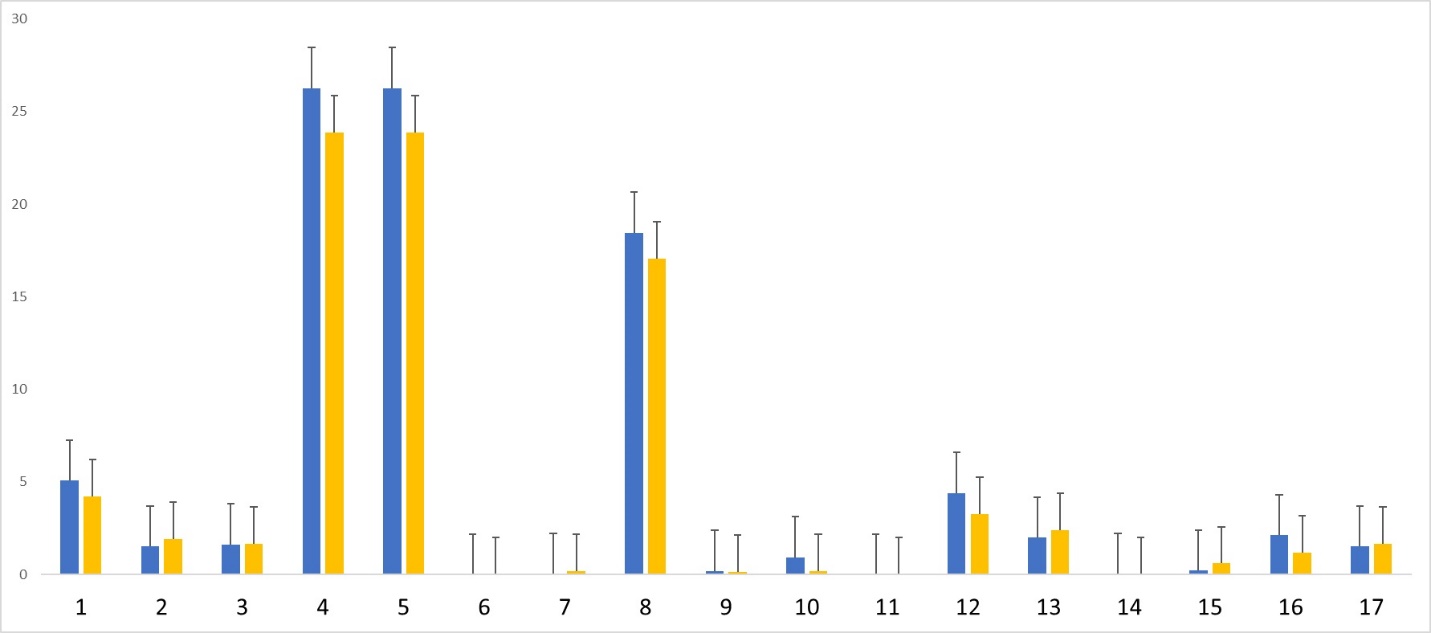


DPP4


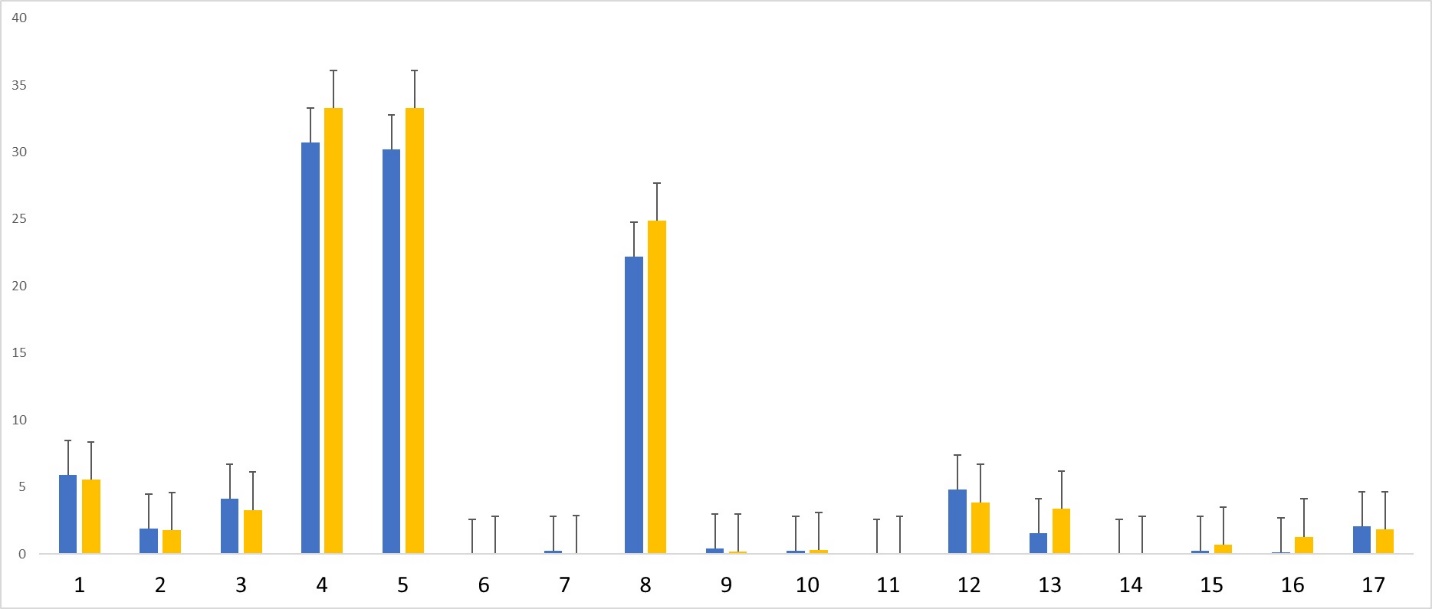


EGFR


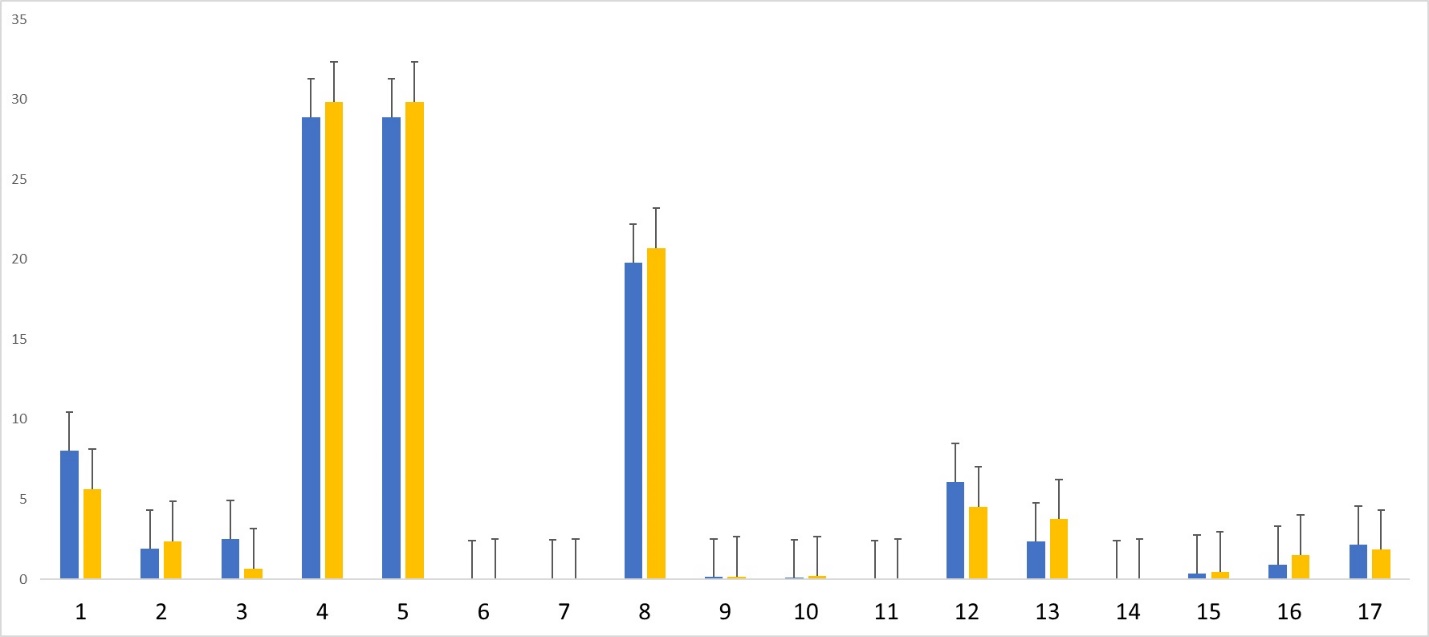


AA2AR


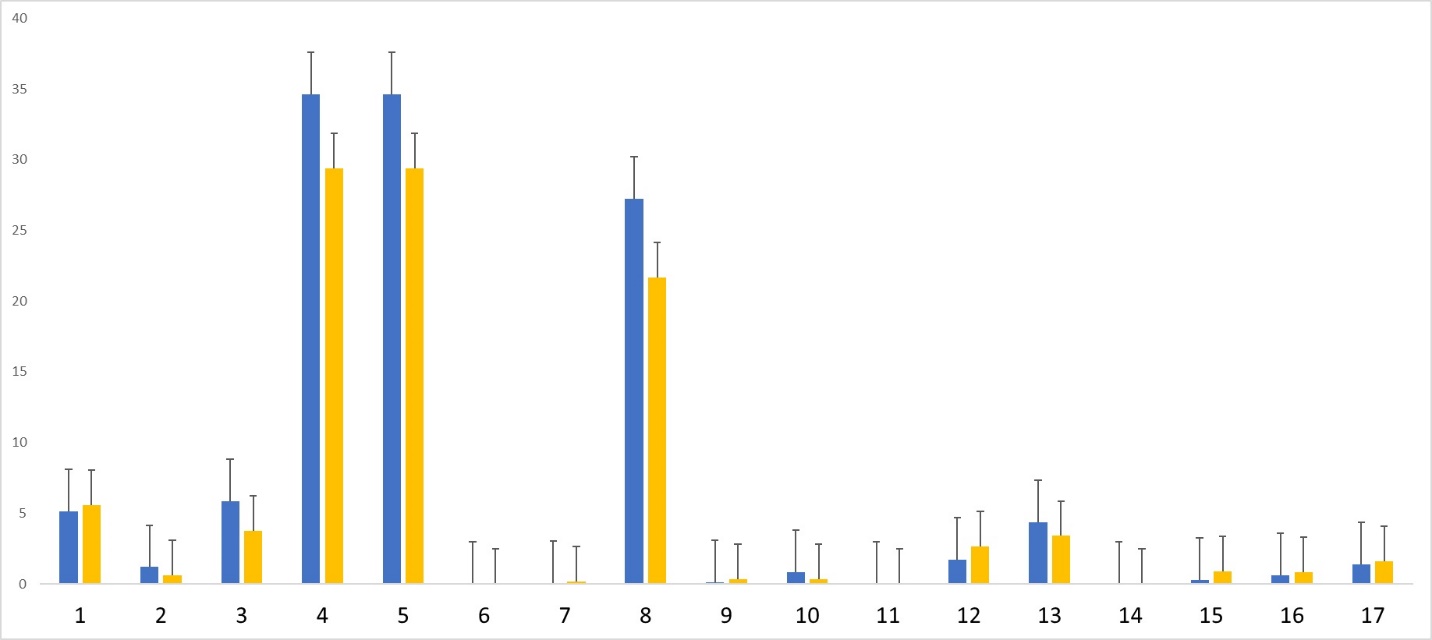


PPARG


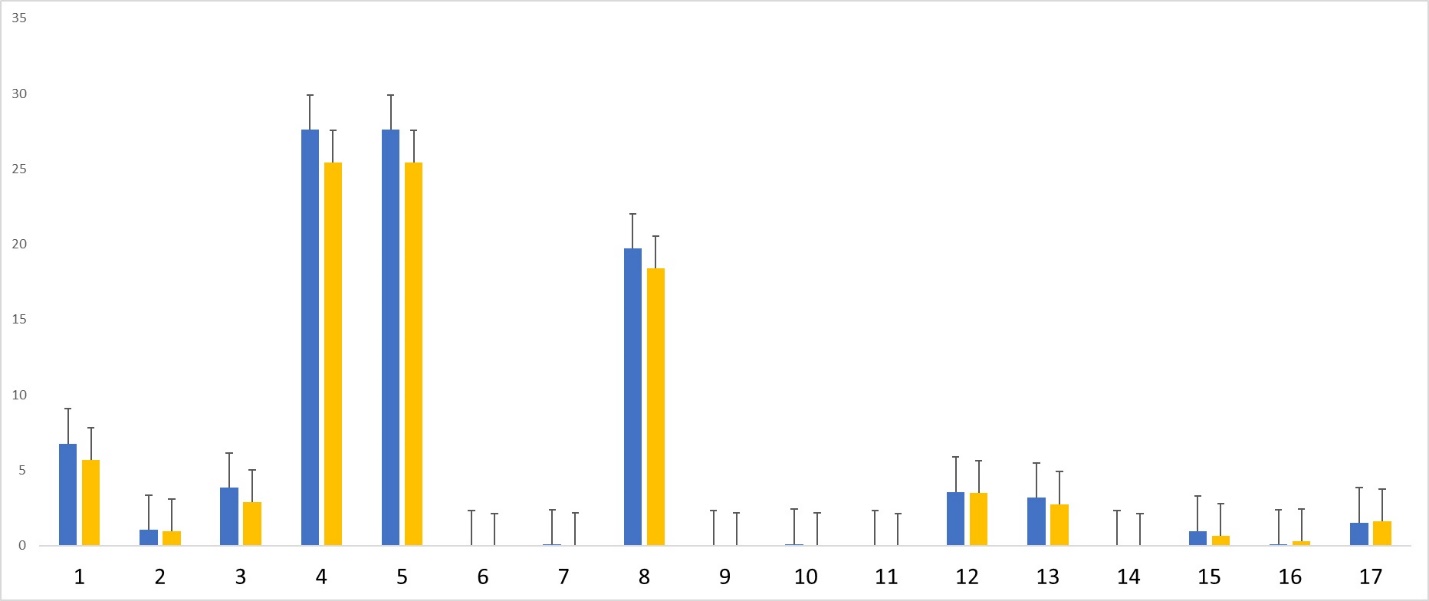


MUV-737


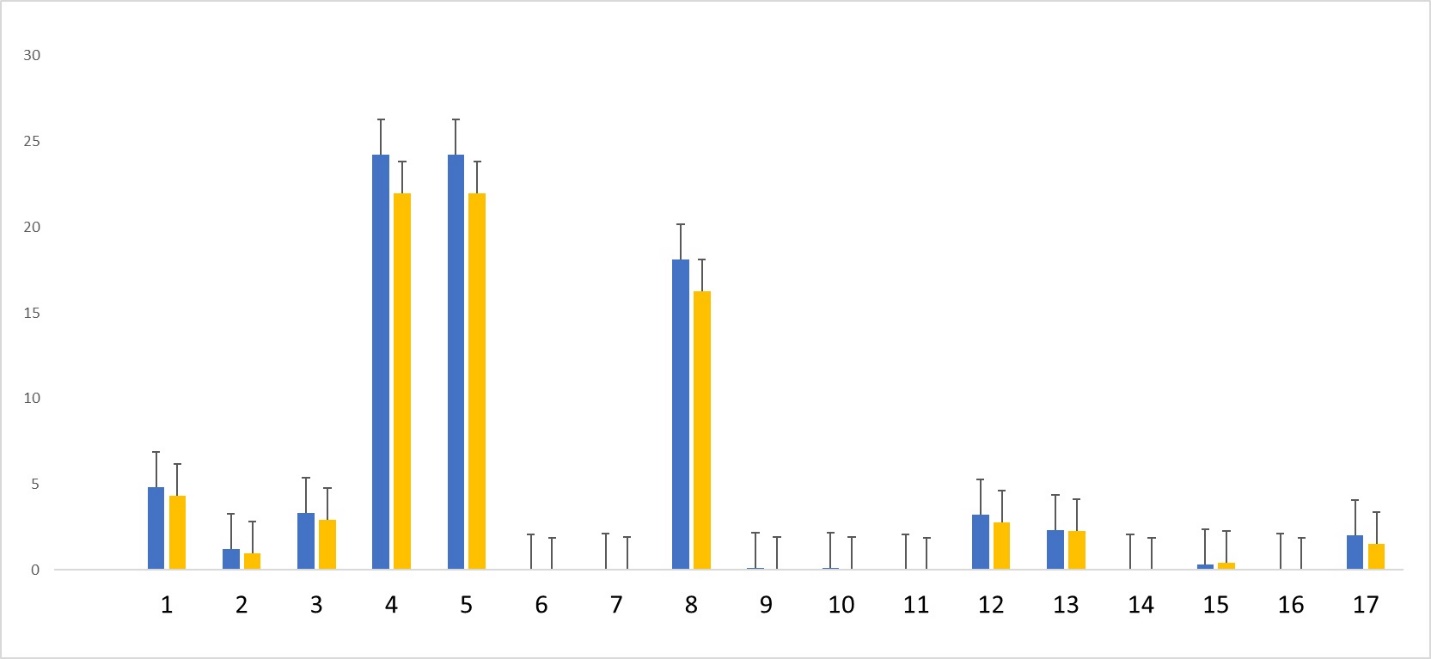


MUV-810


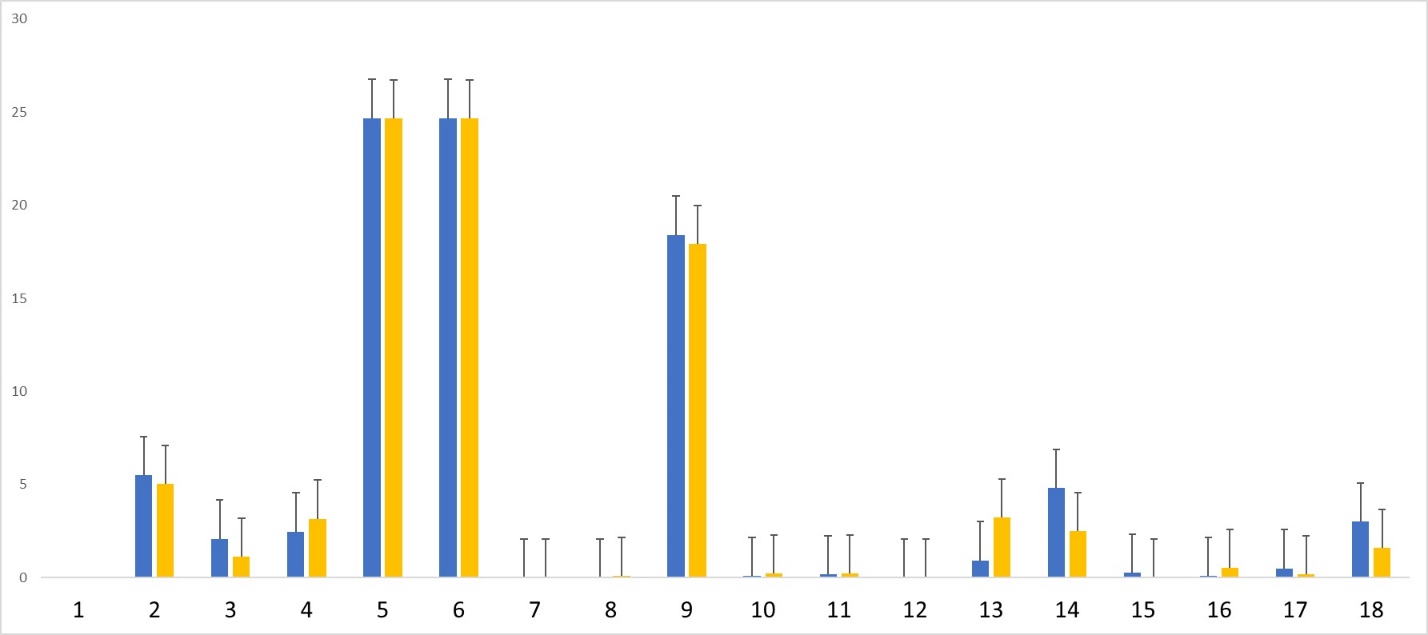


p53


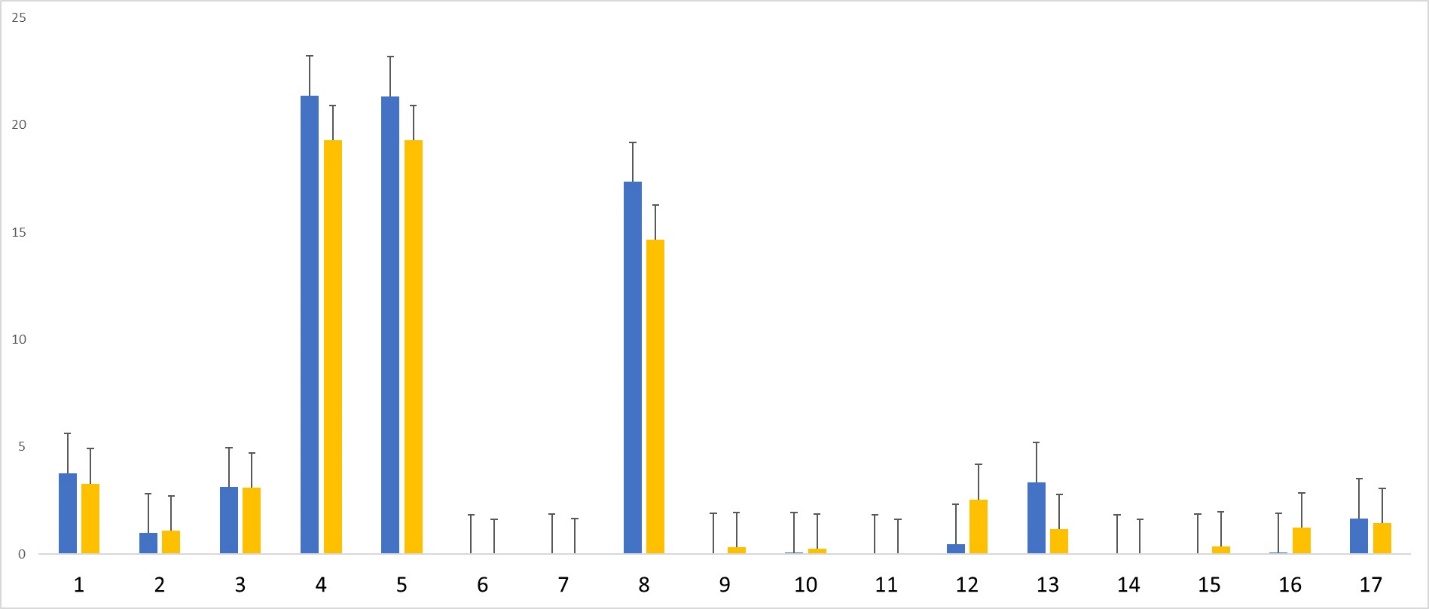


TDP1

**Figure 2, C**


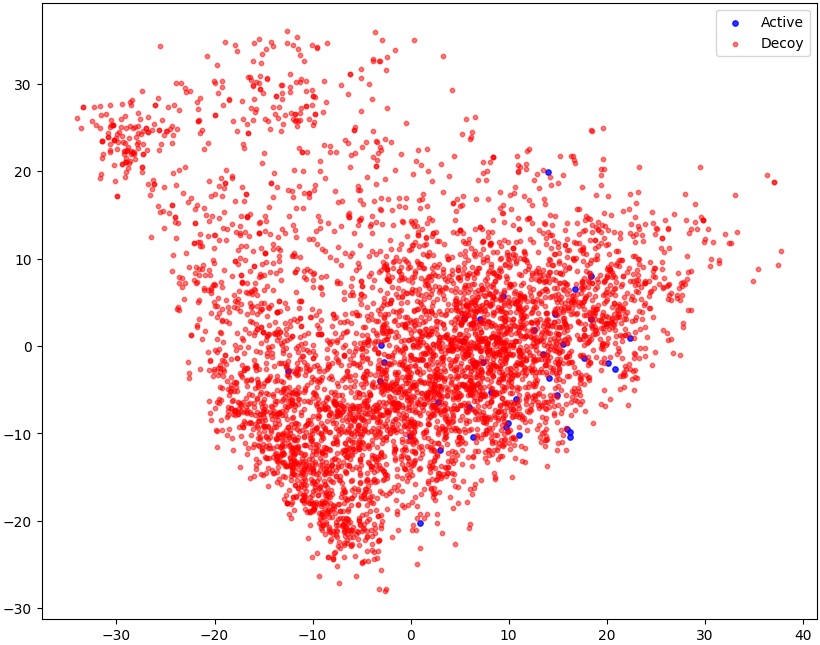


CDK2


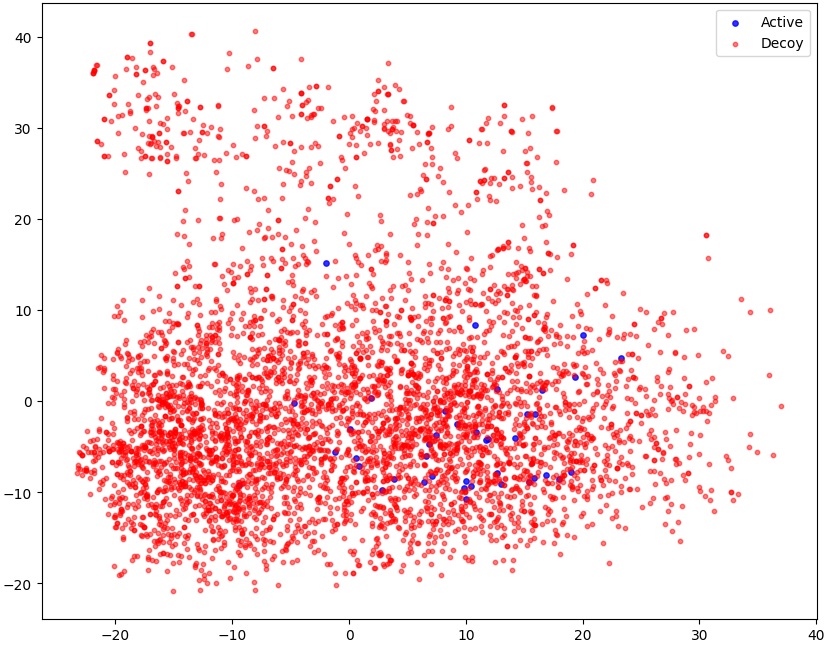


AKT1


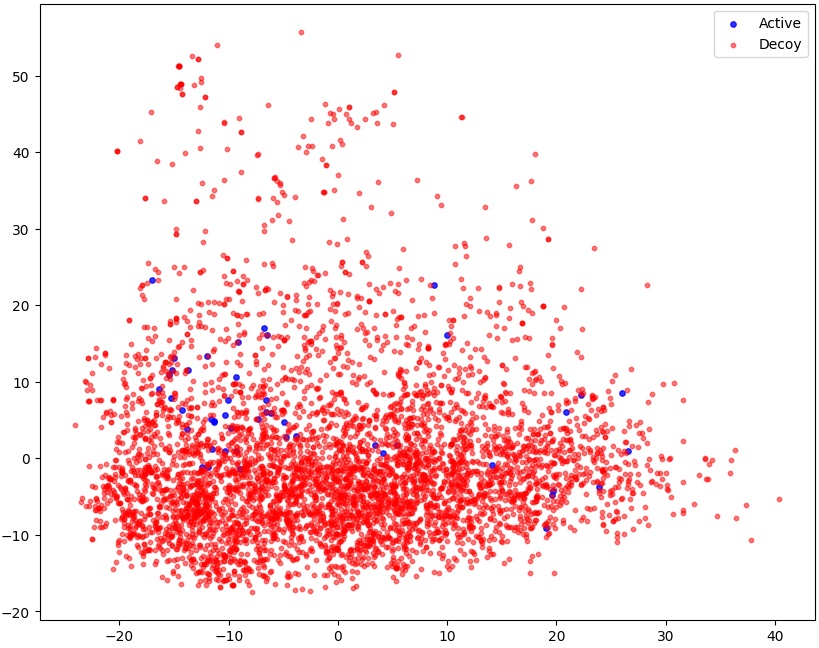


DPP4


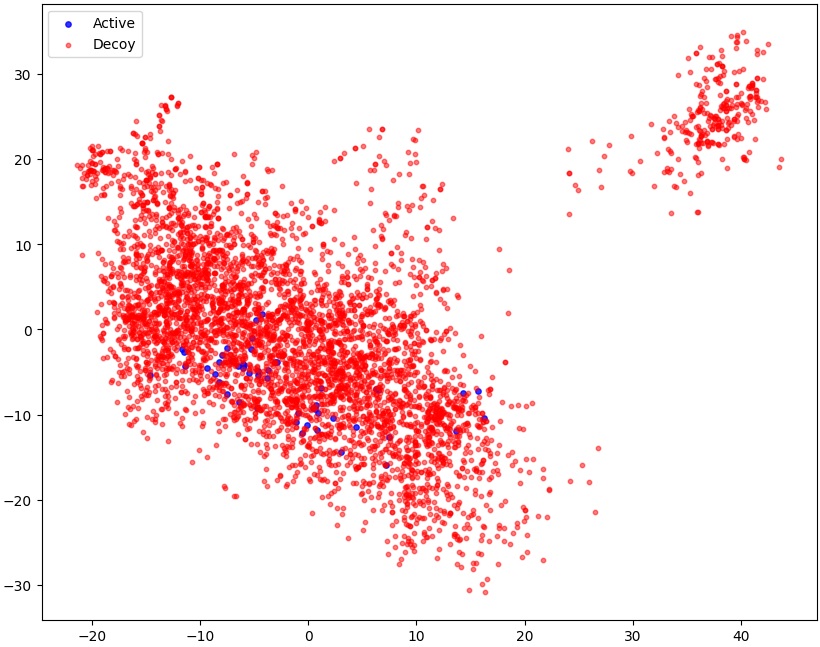


EGFR


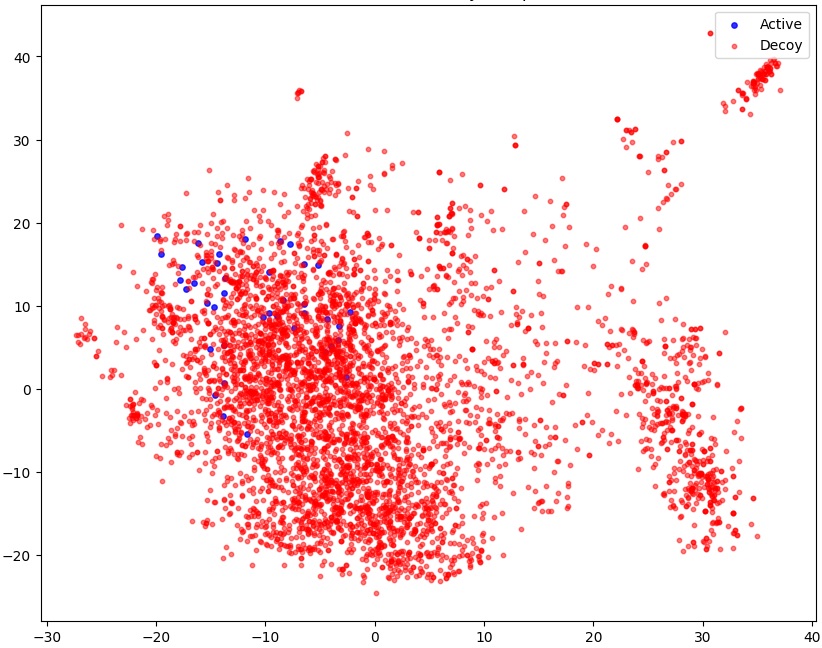


AA2AR


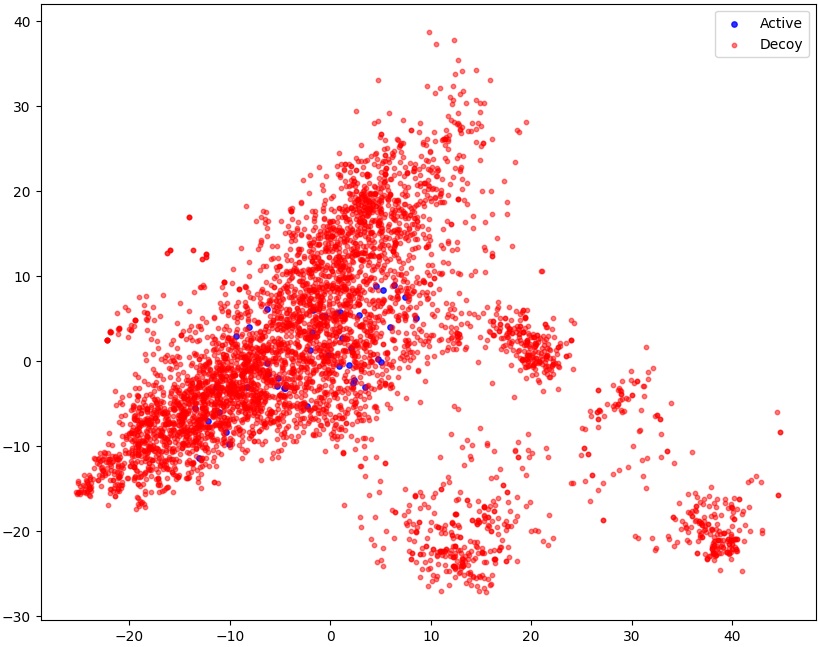


PPARG


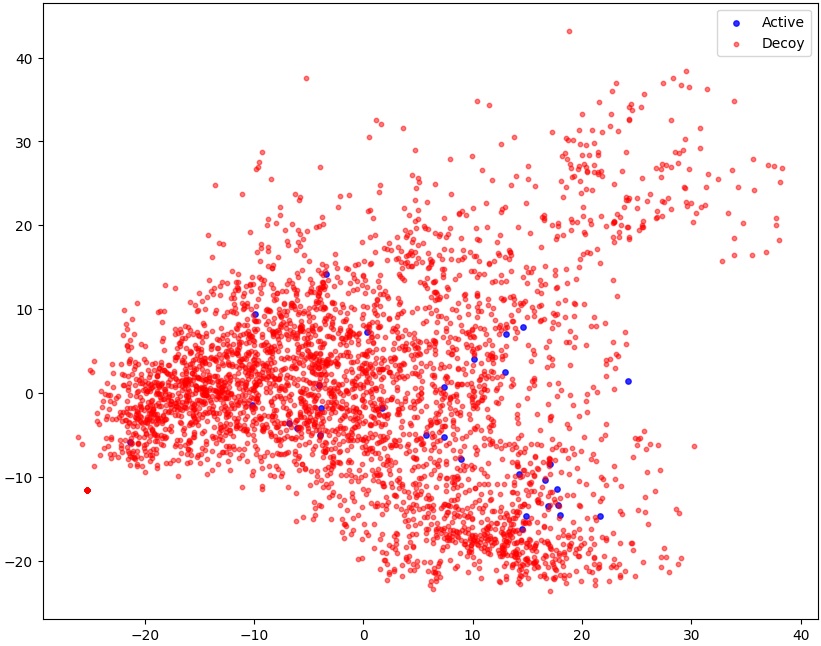


MUV-737


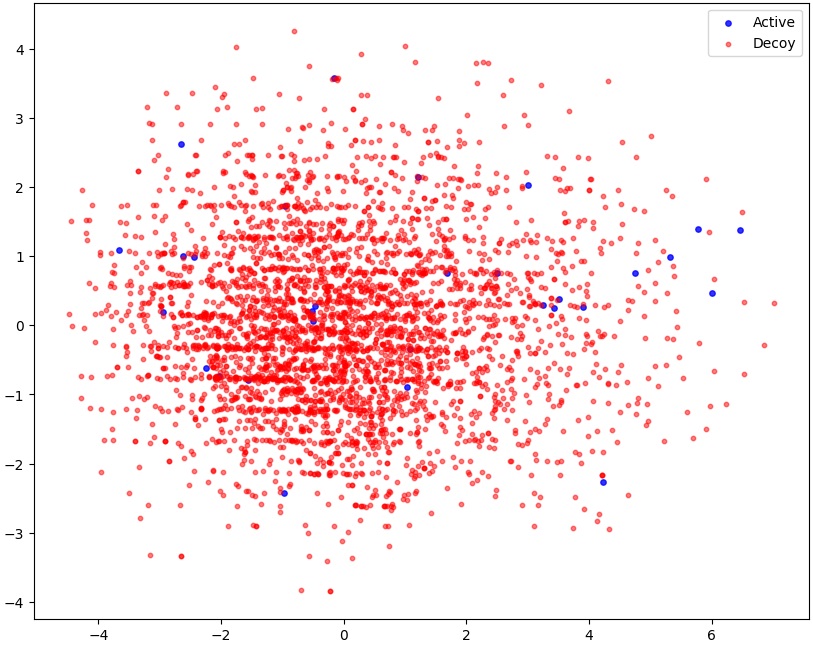


MUV-810


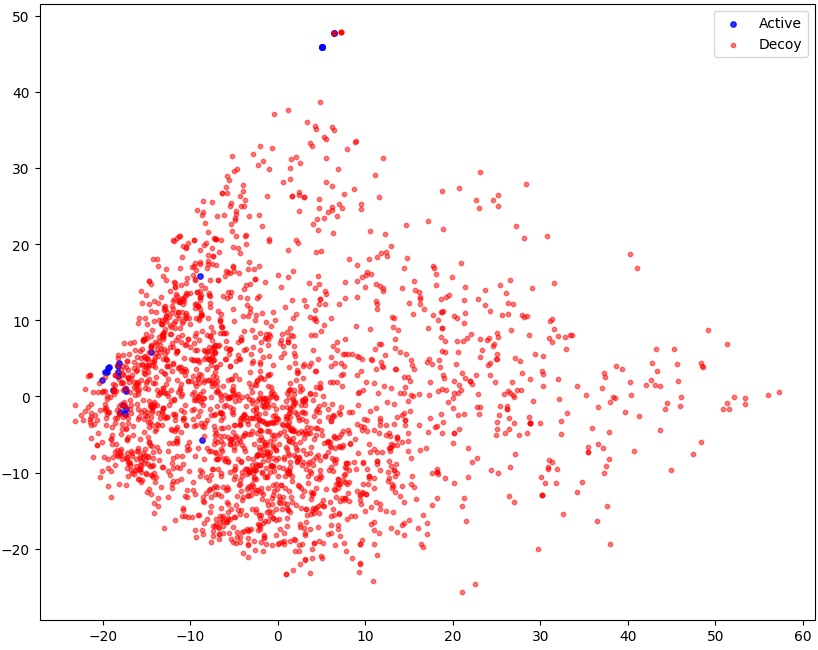


p53


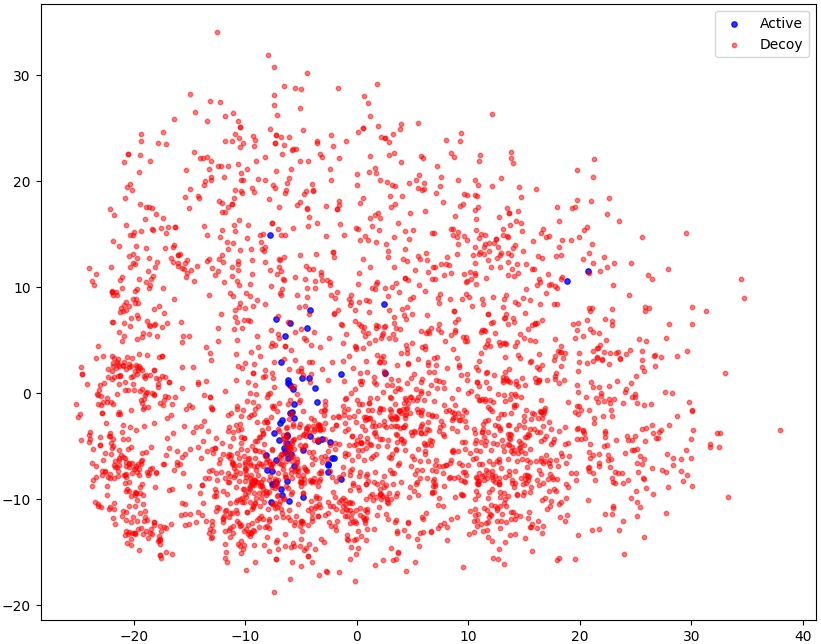


TDP1
